# The Identification of the Metabolism Subtypes of Skin Cutaneous Melanoma Associated With the Tumor Microenvironment and the Immunotherapy

**DOI:** 10.3389/fcell.2021.707677

**Published:** 2021-08-12

**Authors:** Ronghua Yang, Zhengguang Wang, Jiehua Li, Xiaobing Pi, Runxing Gao, Jun Ma, Yi Qing, Sitong Zhou

**Affiliations:** ^1^Department of Burn Surgery and Skin Regeneration, The First People’s Hospital of Foshan, Foshan, China; ^2^Department of Orthopedics, The First Affiliated Hospital of China Medical University, Shenyang, China; ^3^Department of Dermatology, The First People’s Hospital of Foshan, Foshan, China; ^4^Department of Anesthesiology, The First People’s Hospital of Foshan, Foshan, China; ^5^Department of Burns, Nanfang Hospital, Southern Medical University, Guangzhou, China; ^6^Department of Oncology, Affiliated Hospital of Chengdu University, Chengdu, China

**Keywords:** metabolism subtypes, tumor microenvironment, skin cutaneous melanoma, immune signature, mutation landscape, immunotherapy response

## Abstract

Skin cutaneous melanoma (SKCM) is a highly aggressive and resistant cancer with immense metabolic heterogeneity. Here, we performed a comprehensive examination of the diverse metabolic signatures of SKCM based on non-negative matrix factorization (NMF) categorization, clustering SKCM into three distinct metabolic subtypes (C1, C2, and C3). Next, we evaluated the metadata sets of the metabolic signatures, prognostic values, transcriptomic features, tumor microenvironment signatures, immune infiltration, clinical features, drug sensitivity, and immunotherapy response of the subtypes and compared them with those of prior publications for classification. Subtype C1 was associated with high metabolic activity, low immune scores, and poor prognosis. Subtype C2 displayed low metabolic activity, high immune infiltration, high stromal score, and high expression of immune checkpoints, demonstrating the drug sensitivity to PD-1 inhibitors. The C3 subtype manifested moderate metabolic activity, high enrichment in carcinogenesis-relevant pathways, high levels of CpG island methylator phenotype (CIMP), and poor prognosis. Eventually, a 90-gene classifier was produced to implement the SKCM taxonomy and execute a consistency test in different cohorts to validate its reliability. Preliminary validation was performed to ascertain the role of SLC7A4 in SKCM. These results indicated that the 90-gene signature can be replicated to stably identify the metabolic classification of SKCM. In this study, a novel SKCM classification approach based on metabolic gene expression profiles was established to further understand the metabolic diversity of SKCM and provide guidance on precisely targeted therapy to patients with the disease.

## Introduction

Skin cutaneous melanoma (SKCM) is the deadliest type of skin cancer due to its high metabolic and metastatic rates, accounting for more than 80% of skin cancer-related deaths (Guy et al., 2015; Bolick and Geller, 2021). In the past few years, the advancement of immune checkpoint blockade agents has become a pillar of SKCM therapy, remarkably boosting the therapeutic outcomes. However, the response of patients with SKCM to immunotherapy is heterogeneous, with approximately 50% of them experiencing unfavorable responses (Robert et al., 2011; Schadendorf et al., 2015). Therefore, it is imperative to uncover the latent molecular mechanisms of SKCM heterogeneity to develop precise immunotherapies and determine the populations that would benefit the most from them.

Skin cutaneous melanoma is characterized by prominent metabolic plasticity, a feature that results from the activation of oncogenic pathways due to a high frequency of somatic mutations (Alexandrov et al., 2020). SKCM has been classified into four genomic subtypes, BRAF subtype, RAS subtype, NF1 subtype, and triple wild type based on the somatic mutations in these genes and their ratios (2015). These intrinsic oncogenes contribute to the metabolic conversion of SKCM, which leads to a high degree of plasticity and adaptation of melanoma to unfavorable conditions (Ratnikov et al., 2017). Moreover, the transformed metabolic microenvironment can reprogram the function of immune cell subpopulations, allowing melanoma to evade the immune system (Bristot et al., 2020).

A recent proteogenomic research divided patients with SKCM into two subgroups according to whether they responded to immunotherapy against PD-1 or TIL (Harel et al., 2019), namely, the responder and the non-responder subgroups. Differential protein expression analysis found that differences in mitochondrial metabolism were responsible for the response of patients to immunotherapy rather than the treatment protocol. In summary, the heterogeneity of melanoma metabolism is an important reason for the poor efficacy of immunotherapy. Therefore, this study was conducted to categorize SKCM from a metabolic viewpoint to reveal its heterogeneity.

In the present study, we performed a systematic examination of the diverse metabolic signatures of SKCM using a screened metabolic gene based on a non-negative matrix factorization (NMF) clustering algorithm and identified three distinct metabolic subtypes. In this process, The Cancer Genome Atlas (TCGA)-SKCM cohorts were merged into a metadata set for clustering based on the expression of metabolism genes. Additional processed microarray profiles of Gene Expression Omnibus (GEO) SKCM samples were used for external validation. Unsupervised transcriptomic analysis identified three subtypes of SKCM, namely, C1, C2, and C3. In addition, by comparing transcriptomic data from patients with different metabolic subtypes, differentially expressed genes (DEGs) were retrieved. We estimated the prognostic difference, transcriptome features, relationships with metabolic signatures, tumor microenvironment features, immune infiltration, clinical traits, somatic mutation signatures, immunotherapy, and drug sensitivity of the SKCM subtypes, and a comparison was made with previously established classifications. Finally, a 90-gene classifier was used to determine SKCM classification. This research may also provide in-depth insights into tumor–immune cell interactions, showing considerable promise for the clinical therapeutic interventions of patients with SKCM.

## Materials and Methods

### Patients and Samples

Gene expression profiles of SKCM, including TCGA-SKCM (Cancer Genome Atlas Network, 2015), GSE54467, and GSE65904, were obtained from three independent cohorts of patients. In addition, only SKCM samples were retained for further analysis. TCGA-SKCM project was downloaded using the TCGAbiolinks package (Colaprico et al., 2016) and converted to the TPM format for subsequent analysis. The annotation of Ensembl ID for protein-coding mRNAs was transformed to the gene symbol based on the GENCODE gene model (GENCOED27). Then the batch effect from the different datasets was corrected using the ComBat package (Zhang et al., 2020) in the SKCM cohorts. Clinical data regarding the disease, including age, sex, tumor stage, and survival information, were retrieved from TCGA Pan-Cancer Clinical Data Resource (TCGA-CDR), and the clinical characteristics of the TCGA-SKCM patients are shown in Supplementary Table 1, of which only the overall survival (OS) information was obtained for further data processing. In addition, the copy number mutation data of TCGA-SKCM cohorts were downloaded from the GDAC-Firebrowse website^1^.

### Identification of Skin Cutaneous Melanoma Molecular Subtypes by Non-negative Matrix Factorization Clustering

The NMF clustering (Possemato et al., 2011) algorithm was used to cluster the SKCM samples. The 2,752 metabolism-related genes that encode all the well-known human metabolic enzymes and transporters were selected for follow-up screening. First, the metabolism-related genes that were significantly correlated with OS time were subjected to Cox survival regression using the survival R package. Then unsupervised NMF clustering (Gaujoux and Seoighe, 2010) was performed based on the TCGA-SKCM cohort, and validation was performed from the integrated cohorts from GSE54467 and GSE65904 using the same selected candidate genes. K values were chosen where the magnitude of the cophenetic correlation coefficient started to decrease with the optimum number of clusters (Brunet et al., 2004). Next, we evaluated the similarity of subtype classification between independent cohorts based on the expression profiles of mRNAs by employing the class mapping analysis (SubMap) (Gene pattern) method to assess whether the subtypes were analyzed in the training, and validation sets were significantly correlated. Simultaneously, the mRNA expression data of the abovementioned candidate genes were analyzed to verify the subtype distributions using the T-distributed stochastic neighbor embedding (t-SNE) method.

### Gene Set Variation Analysis

Gene set variation analysis (GSVA) (Hänzelmann et al., 2013) is a gene set enrichment method that computes an estimated fraction of certain pathways or signatures of different clusters based on expression profiles. The relevant metabolism (Rosario et al., 2018) and carcinogenesis- (Sanchez-Vega et al., 2018)-relevant pathway gene sets were obtained from previous studies, and the GSVA R package was used to investigate the gene set differences between samples. Subsequently, metabolism gene scores were obtained for differential analysis using the Limma package (Ritchie et al., 2015) in R software, and differentially expressed signatures were screened out with the following threshold (| log_2_FC| > 0.2, adjusted *p* < 0.05).

### Estimation of the Tumor Microenvironment Signatures

The microenvironment cell population counter (MCPcounter) (Becht et al., 2016) was used to estimate the number of infiltrated immune cell populations and two non-immune stromal cell populations (immune cell types: T cells, CD8 + T cells, natural killer cells, cytotoxic lymphocytes, B-cell lineage, monocytic lineage, myeloid dendritic cells, and neutrophils; stromal cell types: endothelial cells and fibroblasts). Furthermore, another approach applied to quantify tumor immune components was the single-sample gene set enrichment analysis (ssGSEA) method (Barbie et al., 2009), which calculates enrichment scores representing the degree to which genes in a particular gene set are coordinately upregulated or downregulated within a single sample. In particular, six immune cell populations, including regulatory T cells (Treg), helper T cells 1 (Th1), helper T cells 2 (Th2), helper T cells 17 (Th17), central memory T cells, and effective memory T cells (Tem), were analyzed using the GSVA R package. In addition, the ESTIMATE algorithm (Yoshihara et al., 2013) was applied to computer immune and stromal scores in different subtypes, thereby, reflecting the features of the tumor microenvironment.

### Characterization of Skin Cutaneous Melanoma Subtypes

After data normalization, differentially expressed genes (DEGs) between different SKCM subtypes were identified using the Limma package (| log_2_FC| > 1 and *p* < 0.01). The gene signature set files “c2.cp.kegg.v6.2. symbols.gmt” and “h,all,v60.2.symbols” were downloaded from the Molecular Signature Database (MSigDB)^2^. GSEA was then applied to investigate the pathway and functional enrichment using the Clusterprofiler R package (Yu et al., 2012) with the significance threshold set to an adjusted *p* < 0.05. Furthermore, previously published molecular classifications of SKCM were predicted using the nearest template prediction (NTP) analysis from gene pattern modules, and then the prediction outcome was compared with our classification.

### Construction and Validation of the Skin Cutaneous Melanoma Gene Classifier

To identify specific genes in the SKCM subtypes, we screened genes with statistically significant differences in the different subclasses according to the following criteria: for adjusted *p* < 0.01 and absolute log_2_ FC > 2. Only the genes with a significantly different expression in all three possible parameters were considered as subclass-specific genes. The top 30 genes with the largest log_2_FC values in each subtype (only genes with log_2_FC > 0 were selected) were further used to generate the prediction models such that a 90-gene subtype classifier was created. Next, we used the NTP algorithm to predict the subclasses of the 90-gene signature in GSE14520 and compared them with the previous classification results derived from the NMF algorithm.

### Prediction of the Efficacy of Each Subtype of Immunotherapy and Targeted Therapy

We used data from patients with melanoma treated with immunotherapy to indirectly predict the efficacy of immunotherapy in melanoma subclasses by measuring the similarity of gene expression profiles between the subclasses determined in this study and those in patients with melanoma based on SubMap analysis (gene pattern). Furthermore, we downloaded and performed SubMap analysis from the genomics of drug sensitivity in cancer (GDSC) database (Yang et al., 2013) to investigate its drug sensitivity.

### Gene Ontology and KEGG Analyses

We performed Gene Ontology (GO) and KEGG enrichment analysis for the different subclasses of differentially expressed genes, where the GO includes biological process (BP), molecular function (MF), and cellular component (CC). GO analysis of differentially expressed genes was conducted using DAVID (Huang et al., 2007; Huang da et al., 2009) (FDR < 0.1) and visualized using the ggplot2^3^ R package and Goplot package (Walter et al., 2015).

We performed the KEGG pathway enrichment analysis using the KOBAS 3.0 database^4^ (Wu et al., 2006) for the integration of DEGs with Ensemble ID in the differential gene list and then obtained pathway enrichment lists where pathways with *p* < 0.05 were considered significantly enriched.

### Mutation Analysis Using a 90-Gene Classifier

The gene mutation and gene copy number data of the 90-gene classifier in 32 TCGA pan-cancer databases were retrieved from the cBioportal (Gao et al., 2013) web portal. The mutation status of these genes in these databases were analyzed, and a bar chart showing the distribution ratio of the 90 genes in TCGA pan-cancer databases by mutation type, fusion, amplification, deep deletion, and multiple alterations were constructed.

### Transcription Factor Prediction

Transcription factors often modulate several metabolic genes that are closely functionally associated. Therefore, we utilized the NetworkAnalyst network platform (Zhou et al., 2019) to analyze and predict the transcription factors that are most likely to regulate the 90 genes included in the classifier and construct a molecular interaction network. The targeted gene–transcription factor interaction network also contains the TF–mRNA–miRNA molecular regulatory network data obtained from the RegNetwork information library.

### Expression of Solute Carrier Family 7 Member 4 in Skin Cutaneous Melanoma and Kaplan–Meier Analysis

RNA-seq data were downloaded from UCSC XENA in TPM format, and GTEx was downloaded from UCSC XENA^5^ and processed by the Toil project (Vivian et al., 2017). The Wilcoxon rank-sum test was used to compare solute carrier family 7 member 4 (SLC7A4) expression in normal TCGA and GTEx skin tissues and tumor samples from SKCM in TCGA. We categorized patients into high and low SLC7A4 expression groups according to the median value of their SLC7A4 expression (TPM format), following which Kaplan–Meier analysis of OS was performed for each group and visualized using the R package “survminer.”

### Patient Tissue Specimen Collection and Immunohistochemistry Validation

Melanoma tissues were collected from 25 melanoma patients of Han Chinese ethnicity from 2015 to 2020. Informed consent was obtained from each patient, and the study was approved by The Foshan Subject Review Board of the First People’s Hospital. Paraffin-embedded tissues were sectioned at 4-mm thickness for immunohistochemistry (IHC) analysis. Antigen retrieval was performed by incubating the samples in citrate buffer (pH 6.0) for 15 min. After blocking with a mixture of methanol and 0.75% hydrogen peroxide, sections were incubated overnight with a primary antibody (SLC7A4, Proteintech, 1:200) followed by incubation with a secondary antibody conjugated with horseradish peroxidase (HRP; goat anti-rabbit IgG, 1:500, Cell Signaling Technology). The sections were washed three times with PBS and incubated with AEC (ZSGB-BIO). Staining was performed as described previously (Zhou et al., 2021). Tissues were examined through the cross-product (H score) of the percentage of tumor cell staining at each of the three staining intensities, and the staining score was graded through the H score as follows: low, H score = 0–100; moderate, H score = 101–200; and high, H score = 201–300.

### Statistical Analysis

All data processing and analyses were performed using Excel (Microsoft) and R software (version 4.0.2). Unpaired Student’s *t*-test was employed for the comparison of two groups with non-normal distribution, and the Mann–Whitney *U*-test was used for the comparison of two groups with non-normal distribution. One-way analysis of variance (ANOVA) and Kruskal–Wallis tests were performed for comparisons among three groups. Contingency table (χ^2^) variables used the χ^2^ test for statistical significance. Survival analysis was performed using the Kaplan–Meier method, and the log-rank test was used for comparison. Univariate Cox proportional risk regression models were used to evaluate risk ratios for univariate analyses. Two-tailed *p* < 0.05 was considered statistically significant.

## Results

### Non-negative Matrix Factorization Determined Three Subtypes of Skin Cutaneous Melanoma

**FIGURE 1:**
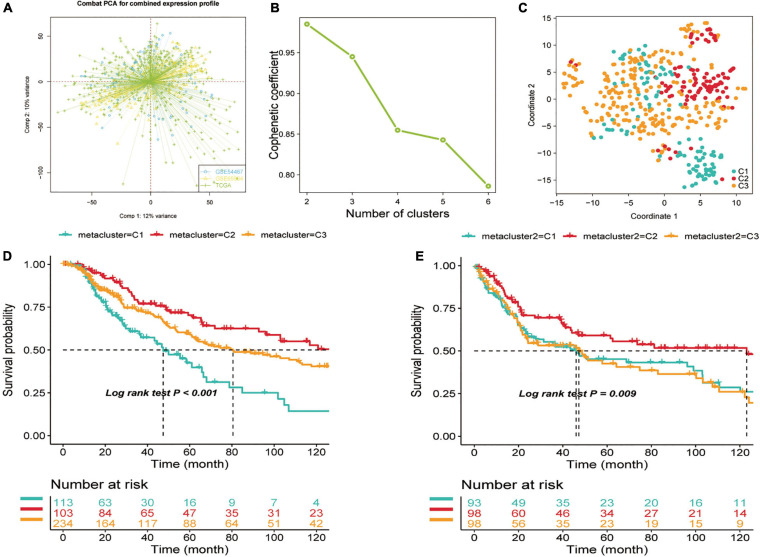
Identification of skin cutaneous melanoma (SKCM) subtypes using non-negative matrix factorization (NMF) consensus clustering in the metadata set. **(A)** Visualization of principal component analysis of combined expression profiles of three cohort data sets. **(B)** Phenotype correlation coefficients for NMF clustering of 816 metabolism-related genes at *k* = 2–5. **(C)** t-SNE analysis rendered in support of classification into three SKCM subtypes. **(D)** Overall survival analysis of three subtypes (C1, C2, and C3) in two independent cohorts—test set (TCGA-SKCM) and **(E)** Validation set (GSE65904, GSE54467). Statistical significance of differences was determined using the log-rank test.

Before conducting the NMF algorithm analysis of SKCM, we first utilized the ComBat algorithm to remove the batch effect for different SKCM cohorts and chart the PCA after batch effect removal (Figure 1A). A total of 2,752 previously reported metabolism-related genes were selected as the basis for NMF analysis. We then adopted univariate Cox regression for metabolism-related genes in the metadata set to identify prognostic genes associated with OS (*p* < 0.1), and a total of 517 candidate genes were obtained. We then extracted TCGA data expression profiles of SKCM, clustering the 517 candidate genes using the NMF clustering algorithm, and the non-negative matrix decomposition (NMF) with two to six clusters was plotted (Figure 1B). The best clustering number *k* value was established by computing the clustering correlation coefficient, and *k* = 3 was regarded as the optimal clustering number. The consensus NMF was performed again with several decompositions of 3, defining three subtypes C1 (*n* = 113), C2 (*n* = 103), and C3 (*n* = 234). To validate the clustering subtypes, we performed t-SNE dimension reduction to reduce the feature dimensionality for all samples of metabolic genes in the expression profile of the test TCGA dataset and showed the different two-dimensional distribution pattern plots for the three types of samples (Figure 1C), and the clinical characteristics of TCGA-SKCM are shown in Supplementary Table 1. We also found that our subtypes were largely consistent with the two-dimensional t-SNE distribution pattern. The same consensus NMF for the same set of metabolic genes was performed for the consolidated validation set (GSE54467 and GSE65904), and three classes C1 (*n* = 93), C2 (*n* = 98), and C3 (*n* = 98) were obtained. Finally, the subclass algorithm was used to identify the subtype matching model of TCGA and validation sets, and it was decided that TCGA-C1 = GEO-C1, TCGA-C2 = GEO-C3, and TCGA-C3 = GEO-C2. We also utilized survival information from the three cohorts to conduct a subtype survival analysis of the SKCM subset. In TCGA-SKCM cohort, the results showed a significantly higher OS in C2 than in C1 and C3 (log-rank test *p* < 0.0001, Figure 1D), and the same survival differences were also verified in the GEO validation dataset (GSE65904 and GSE54467). Similar survival outcomes were observed; however, the prognosis of C2 in the validation set worsened, and the median survival time was more similar to that of C1 (Figure 1E).

### Association of Skin Cutaneous Melanoma Subtypes With Metabolism-Related Signatures

**FIGURE 2:**
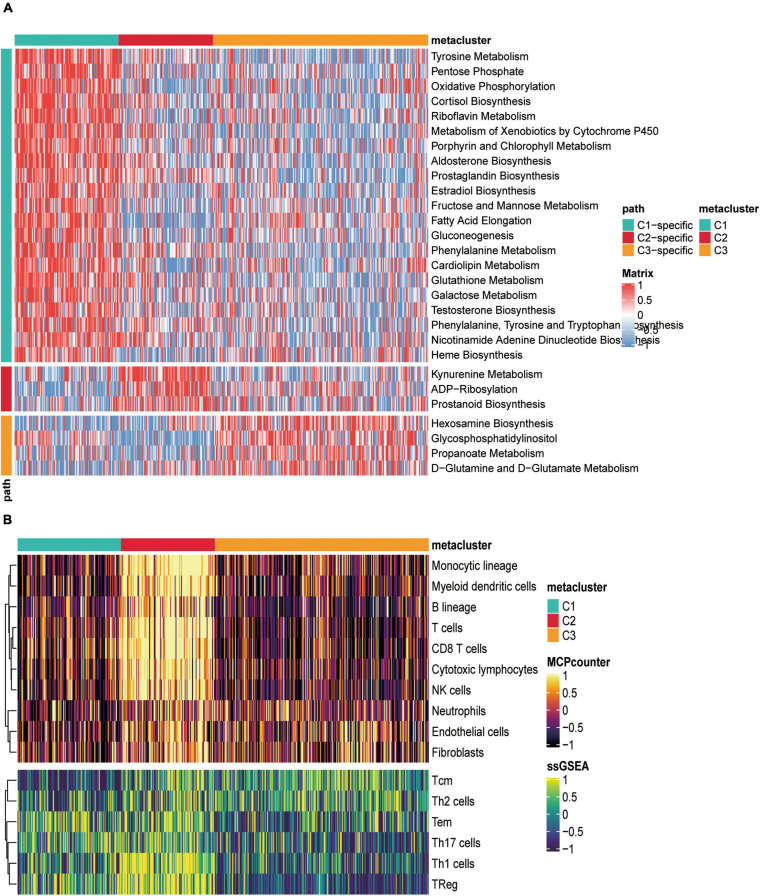
Correlation between metabolically relevant features and immune infiltration in SKCM subtypes. **(A)** Heat map of specific metabolism-related signatures in three subtypes. **(B)** The heat map depicted the richness of immune and stromal cell populations in C1, C2, and C3 by MCPcounter and ssGSEA.

Considering that the SKCM subclass classification was based on metabolism-related genes, we further investigated whether different metabolic signatures are present in the different subclasses. First, we scored the metabolic pathways (gene sets were acquired from the reported paper) using the GSVA R package in the subtype cohorts. In TCGA-SKCM cohort, GSVA enrichment scores of the metabolic pathways were estimated, and a cross-group Limma difference test was performed using two groups of “subtype n vs. other subtypes” to confirm subtype-specific differential metabolic pathways; the screening standard for GSVA enrichment was | logFC| > 0.2, adjusted *p* < 0.05, and heat maps were constructed for visualization (Figure 2A).

The heat map showed that C1, C2, and C3 had 21, 3, and specific metabolic signatures, respectively. Among them, C1 demonstrated a distinct metabolic signature, with 21 metabolic pathways significantly upregulated. Both C2 and C3 were significantly downregulated in these pathways. These results demonstrated that each subtype was enriched in unique metabolic pathways and had dissimilar metabolic levels.

**FIGURE 3:**
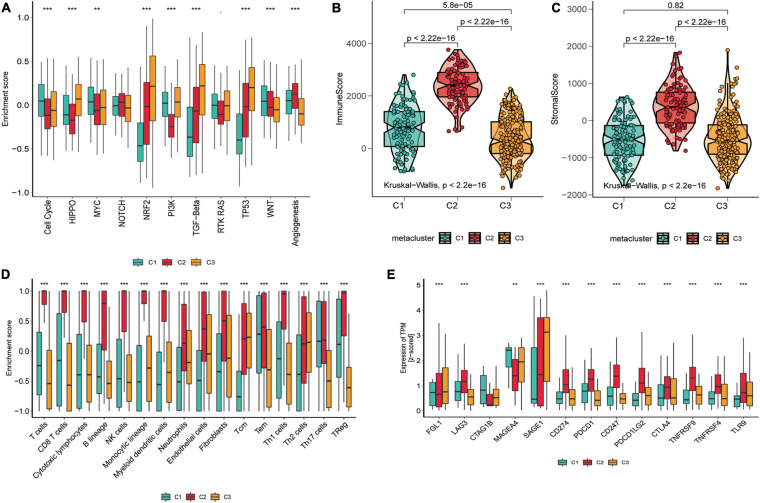
Tumor microenvironment signatures of three subtypes in the TCGA-SKCM. **(A)** Box plots of characteristic marks discriminating different subtypes of SKCM progression-related features. Box-line plots of the immune score **(B)** and stromal score **(C)** estimate three subtypes. For the box-line plot, the lines in the box represent the median, the bottom, and top of the box, which are the 25th and 75th percentiles (interquartile range), and the vertical lines represent 1.5 times the interquartile range. Statistical differences were compared by the Kruskal–Wallis test, and *p*-values are marked with an asterisk above each box line plot (ns indicates no significance, **p < 0.01, ****p < 0.0001). **(D)** The boxplot showed the accumulation of immune and stromal cell populations discriminated by different subtypes. **(E)** Expression level (normalized transcripts per million) of 14 immune checkpoint genes in three SKCM subtypes. The difference was verified statistically through the Kruskal–Wallis test, and the p-values are noted with asterisks at the top of each boxplot (ns stands for no significance, *p < 0.05, ***p < 0.001).

We also counted the GSVA enrichment points of carcinogenesis-related pathways and plotted a box line among groups. To further investigate their subtype characteristics, we selected a collection of 11 carcinogenesis-related pathways and quantified them using the GSVA algorithm. The results showed significant between-group differences among the three subgroups in cell cycle, HIPPO, MYC, NRF2, PI3K, TGF-β, TP53, WNT, and angiogenesis, which are carcinogenic signaling pathways (Figure 3A), indicating a close connection between our subtypes and carcinogenesis. The results revealed that C1 had a significantly strong cell cycle and WNT signature than C2 and C3, C2 displayed increased expression of components of the angiogenesis pathway, and C3 was particularly enriched in the HIPPO, NRF2, PI3K, TGF-β, and TP53 pathways.

### Correlation Between Skin Cutaneous Melanoma Subtypes and Immune Infiltration

As significant differences in immune scores were detected between SKCM subtypes, we surveyed immune cell infiltration in SKCM subtypes to assess their immunological landscape. We utilized the MCP-counter and ssGSEA algorithms to compute the abundance of 16 immune infiltration cells and presented them in an immune heat map (Figure 2B). We also plotted box plots of inter-group differences in immune cells, and the results displayed significant inter-group differences in all types of immune cell populations among the three subtypes (Figure 3D). Notably, the box plots revealed that C2 showed a significantly higher enrichment immune score than C1 and C3 in almost all immune cells, except for Th17 cells, in which both C1 and C2 had significantly higher Th17 cell enrichment scores than C3. Of these results, C2 was enriched with more immune cells, which is consistent with the finding that C2 had the highest immune score among the three subtypes. With the current widespread use of immune checkpoint inhibitors (ICIs) in clinical trials and for the treatment of advanced melanoma (Woods et al., 2016), we explored the correlation between the expression of 13 classically targeted immune checkpoint genes in these subtypes, which are currently based on immunotherapy inhibitors in clinical trials or licensed for certain cancer types. The results showed significant differences between groups, while C2 displayed a higher expression of nine immune checkpoint genes than C1 and C3, except for FGL1, CTAG1B, and MAGEA4 (Figure 3E).

### Relevance of Skin Cutaneous Melanoma Subtypes to the Clinical Characteristics of Patients and in TCGA and GEO Datasets

**FIGURE 4:**
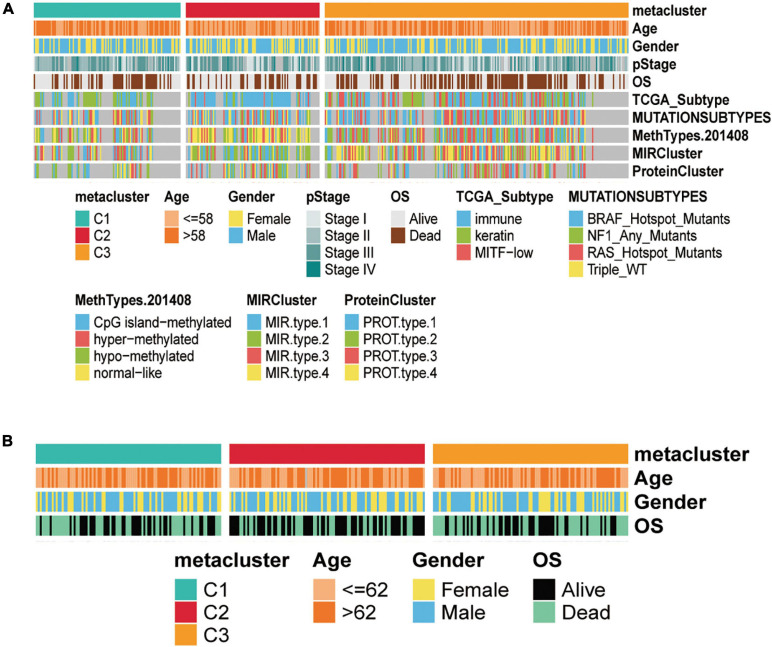
Clinical characteristics of three subtypes in the TCGA-SKCM and GSE14520 cohorts. **(A)** Correlation of C1, C2, and C3 subtypes with clinical features and previous SKCM subtypes in the TCGA cohort. **(B)** Correlation of our classification with clinical features in the validation GSE14520 set cohorts.

To investigate the associations between subtypes and clinical characteristics, we analyzed the clinical tumor pathology variables associated with subtypes based on TCGA-SKCM (Figure 4A) and GEO validation set (Figure 4B) cohorts to construct the clinical information heat map of subtypes. The results showed an independence test for discrepancies between clinicopathological characteristics and metabolic subtypes. Furthermore, we matched this metabolic classification with the previously reported subtypes of SKCM in the literature, including mutation subtypes (BRAF, NF1, RAS, and Triple-WT), MethTypes (CpG island-methylated, Hyper-methylated, Hypo-methylated, and Normal-like), the MIR cluster (four types), the Protein Cluster (four classes), and the classification of TCGA (Immune, Keratin, and MITF-low) (Figure 4A and Supplementary Tables 2, 3). In TCGA-SKCM, the C1 subtype was significantly correlated with protein cluster 2 (*p* = 0.006), MIR type 3 (*p* < 0.001), hypomethylated type (*p* < 0.001), and keratin type (*p* < 0.001). The C2 subtypes associated with protein cluster 1 (*p* = 0.006), MIR type 2 (*p* < 0.001), normal-like methylated type (*p* < 0.001), and immune type (*p* < 0.001). The C3 subtype was related to protein cluster 3 (*p* = 0.006), MIR type 3 (*p* < 0.001), hyper-methylated type (*p* < 0.001), and immune type (*p* < 0.001).

### Association of Skin Cutaneous Melanoma Subtypes With the Tumor Microenvironment

To further evaluate whether the subtypes were associated with the tumor microenvironment, we estimated the immune and stromal scores using the ESTIMATE algorithm for each group and constructed box-violin plots (Figures 3B,C). The results showed statistically significant differences in immune scores between the three groups, while the stromal scores were not statistically significant among the three groups. C2 had the highest immune score among the three groups.

**FIGURE 5:**
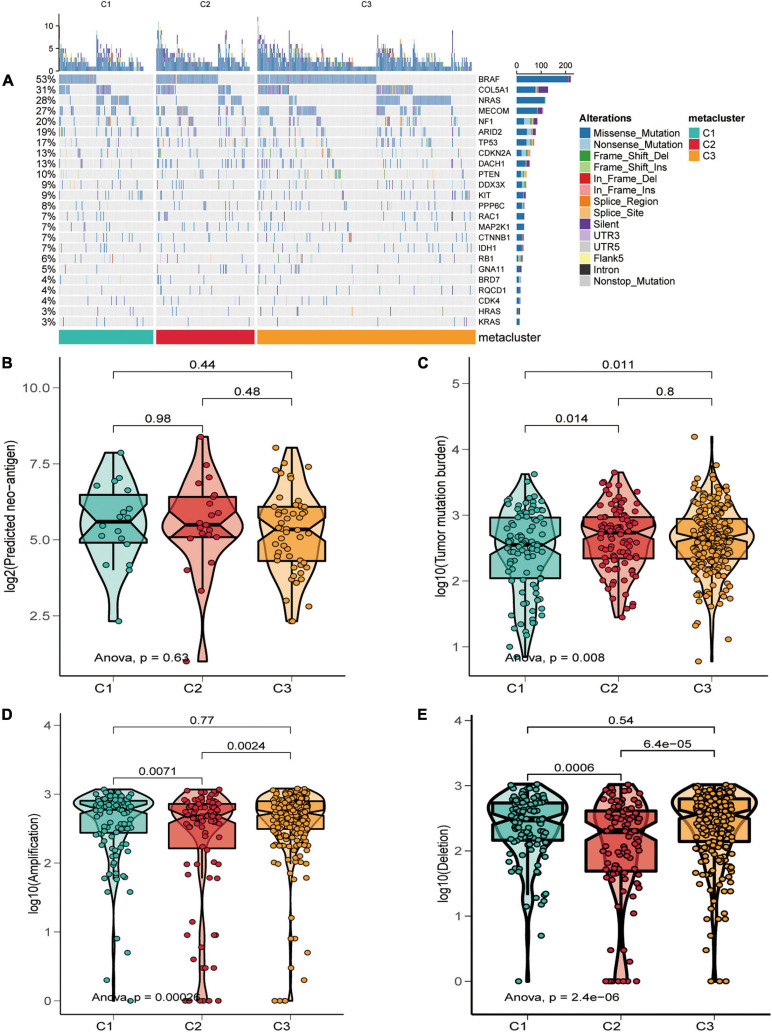
Relationship of SKCM subtypes with tumor mutation burden characteristics. **(A)** Driver-type oncogenic mutations based on TCGA-SKCM typing with intra-group aggregation waterfall plots. **(B–E)** Violin plots of the number of mutation-predicted neoantigens **(B,C)** and copy number aberrations **(D,E)** box lines for SKCM subtypes. Wilcoxon rank-sum test was used to compare statistical differences (ns indicates not significant).

The melanoma mutation landscape has been demonstrated to lead to alterations in the tumor microenvironment (Nassar and Tan, 2020) and immunotherapeutic response (Havel et al., 2019). Next, we investigated whether somatic mutation frequencies varied across SKCM subtypes and examined the mutation patterns in these subtypes. We applied the maftools package (Mayakonda et al., 2018) to estimate TCGA-SKCM-driven gene mutations and mapped waterfall plots of clusters within each group. High frequencies of mutations were observed for BRAF, COL5A1, NRAS, MECOM, NF1, ARID2, TP53, and CDKN2A in SKCM subtypes (Figure 5A and Supplementary Table 4). We then classified the total number of mutations and the predicted neoantigens (Figure 5B). The tumor mutation burden (TMB) was calculated for each metabolic subtype (Figure 5C), and box-line violin plots were constructed separately. Subsequently, we determined the frequency of amplification (Figure 5D) and deletion (Figure 5E) for the three subtypes using online GISTIC 2.0 analysis (Mermel et al., 2011) to construct box-line violin plots of amplifications vs. deletions for each subtype.

**FIGURE 6:**
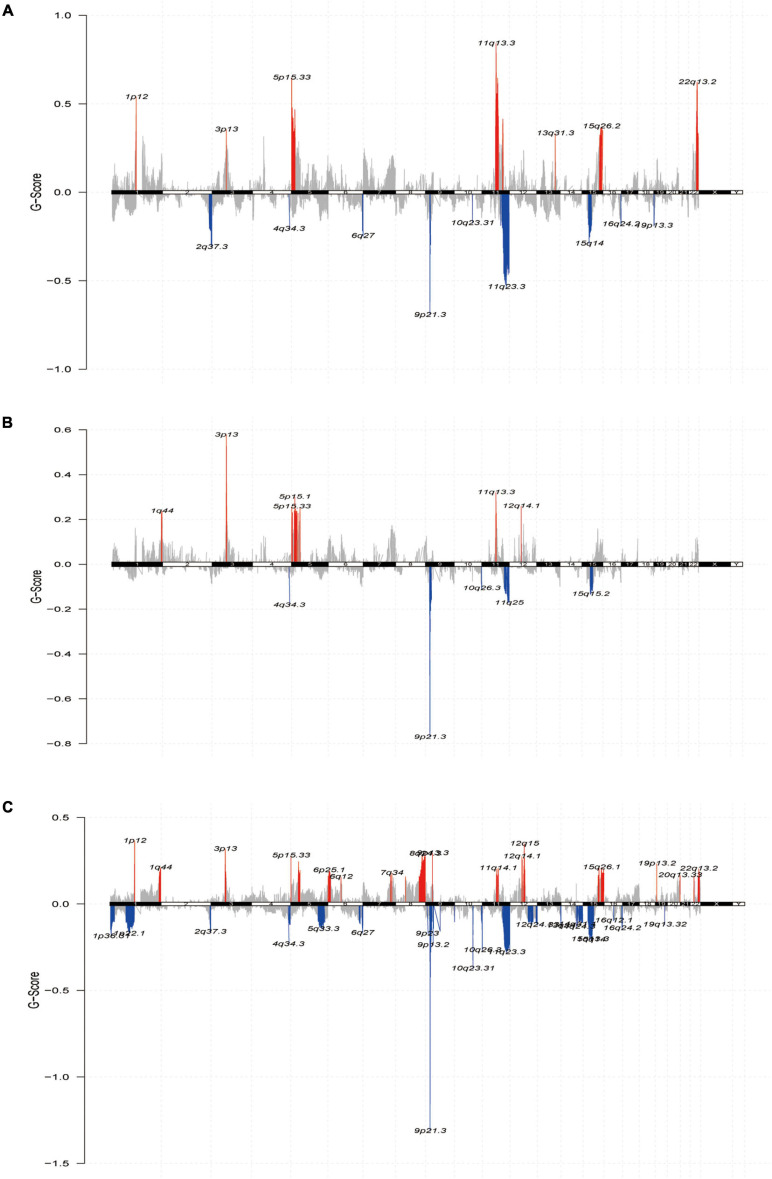
The landscape of somatic copy-number alterations in the three subtypes. **(A–C)** Cytoband showed the genomic copy-number change from three subtypes. Amplification (red) and deletion (blue) of each regional peak are shown and highlighted.

Finally, we further reviewed the chromosomal segment values of the three subtypes to ascertain whether there were significant copy number alterations by performing online GISTIC2.0 analysis on genePattern^6^ to map the copy number change cytoband of each subtype. Cytoband revealed an overall description of the copy number variation in each subgroup (red representing gains and blue representing losses) (Figures 6A–C). As expected, our profiling demonstrated that significant copy number alterations emerged in the three subtypes, including those observed in the chromosomal region of 9p21.3 (CDKN2A) (Ghiorzo et al., 2006); amplification at 11q13.3 (CCND1) (Gibcus et al., 2007) in C1 and C2; amplification at 22q13.2 (TOB2) (Thanasai et al., 2006) in G1 and G3, in which TOB2 was significantly amplified in C1; and a major amplification at 5p13.3 (TERT) (Peifer et al., 2015) in the C1 subtype. While the cytobands for C1 and C3 displayed more regional amplifications and deletions than C2, this could also be an essential explanation for the superior prognosis of C2 over the C1 and C3 subtypes. Thus, the alteration of copy number may be a dominant mechanism responsible for the differences in metabolism and prognosis among the three subtypes.

### Ninety-Gene Classifier and Performance Validation

**FIGURE 7:**
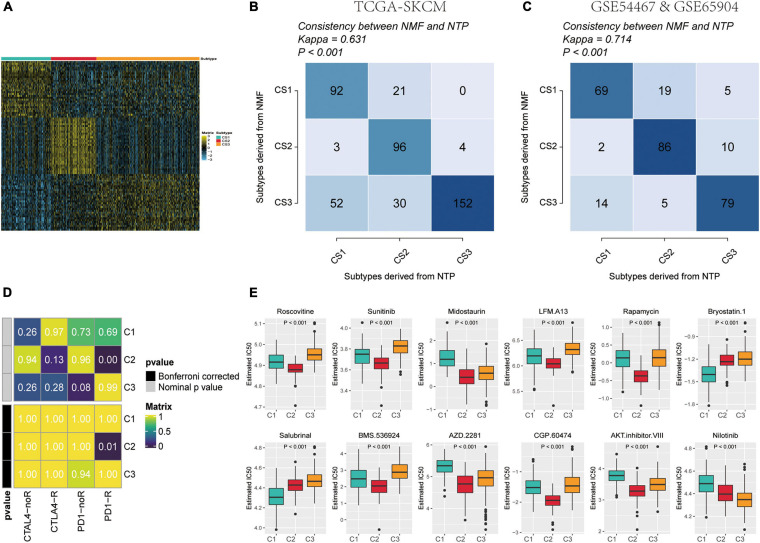
Identification of predictive metabolism-gene classifier and prediction immunotherapeutic response. **(A)** The heat map exhibited the expression level of the 90-gene classifier in C1, C2, and C3. **(B)** Consistency of SKCM metabolism subtype predictions between the 90-gene classifier and the original NMF-based predictions in TCGA-SKCM. **(C)** Consistency of SKCM metabolism subtype predictions between the 90-gene classifier and the original NMF-based predictions in GSE54467 and GSE65904. **(D)** C2 is likely sensitive to the PD-1 receptor inhibitor (nominal *p* = 0.01) by SubMap analysis. **(E)** Top 12 box plots of predicted IC50 values based on GDSC database drugs in three subtypes of TCGA-SKCM dataset.

To build a subtype classifier for clinical application, we further selected subtype-related signature genes. The results of the Limma differential expression analysis were based on the whole expression map “subtype n vs. other subtypes.” Using MOVICS (Lu et al., 2020) package analysis, we retrieved the top 30 genes specifically upregulated for each metabolic subtype as biomarkers and then constituted clinical models and plotted correlation heat maps (Figure 7A and Supplementary Table 4). Consequently, we derived a 90-gene classifier and executed a consistency test by operating the NTP algorithm to predict the metabolic subtype attribution of each sample in the TCGA cohort as well as the GEO test set cohort, and plotted the heat map of the true subtype if it matched the predicted subtype (Figures 7B,C). The results were largely consistent between NMF and NTP in the three different subtypes of the test and validation sets (κ = 0.631 *p* < 0.001, κ = 0.714 *p* < 0.001), indicating that this 90-gene signature could be replicated to identify the metabolic classification of SKCM.

### Specific Sensitivity of Skin Cutaneous Melanoma Subtypes to Immunotherapy and Potentially Targeted Therapies

On the one hand, the different patterns of immune infiltration and expression levels of immune checkpoint genes in different SKCM subtypes suggest the need to further investigate the possibility of an immunotherapeutic response. Therefore, we matched the expression profiles of the three subtypes using a subclass algorithm to ascertain the degree of similarity of TCGA metabolic subtypes to the response profiles of 47 patients with melanoma receiving immunotherapy (Hoshida et al., 2007; Roh et al., 2017). The results indicate that the TCGA-C2 subtype was more likely to be responsive to immunotherapy with anti-PD1 treatment (Figure 7E) (Bonferroni correction, *p* = 0.01).

On the other hand, to identify potential anti-melanoma drugs that are associated with ICIs, we attempted to find potentially sensitive and selective chemotherapy drugs using the GDSC drug sensitivity database. We compared drug sensitivity for more than 100 drugs in the GDSC database, and the top 12 differential response drugs were plotted and listed according to the Kruskal-Wallis test (Figure 7D). We detected a significant difference in the estimated IC50 values between the three subtypes and found that C2 may be more sensitive to chemotherapeutic drugs.

### Functional and Pathway Enrichment Analysis of the 90-Gene Classifier

Gene Ontology and KEGG enrichment analyses were conducted for the three group DEGs using the DAVID database, and GO enrichment analyses were performed for each of the three GO categories: BP, MF, and CC. The ggplot2 package was used for visualization, and the top 10 enrichment results are shown (Supplementary Figure 1 and Supplementary Table 6–8). The GO enrichment results of the three groups of DEGs were mainly enriched in the metabolism process, immune-specific BP, protein binding, cell cycle, DNA damage and repair, and cancer-associated biological processes. By taking the intersection of the enrichment analysis results of the three groups of differential genes in the KOBAS database, we identified six KEGG pathways related to oncogenesis development and metabolism for presentation. The red dots represent the upregulated enriched genes, mainly enriched in apoptosis, cell cycle, HIF-1 signaling pathway, human T-cell leukemia virus 1, cancer infection pathway, PI3K–Akt signaling pathway, and carcinogenesis biological pathway (Supplementary Figure 2 and Supplementary Table 9).

### Mutation Analysis of the Gene Classifier in TCGA Pan-Cancer Database

Based on the gene mutation and gene copy number data of the 90-gene classifier in 32 TCGA pan-cancer databases retrieved from the cBioportal web portal, the mutation rates of the genes in the classifier were analyzed, and the distribution ratio of each mutation type of the gene classifier in each pan-cancer tumor, including gene mutation, fusion, amplification, deep deletion, and multiple alterations, is shown in a bar chart (Supplementary Figure 3A). Among them, the mutation rate exceeded 50% in most tumor cancer types in TCGA, and the mutation rate was higher in lung squamous cell carcinoma, esophageal adenocarcinoma, stomach adenocarcinoma, SKCM, and bladder urothelial carcinoma, with an alteration frequency of more than 80%.

### The TF–mRNA–miRNA Network Construction

Many closely related genes are often subject to simultaneous regulation by specific transcription factors; therefore, we utilized the NetworkAnalyst network platform to predict the transcription factors most likely to regulate the 90 genes in the classifier by analyzing them and constructing a TF–mRNA–miRNA molecular interaction regulatory network (Supplementary Figure 3B).

### Expression Signature, Prognostic Value, and Preliminary Experimental Validation of Solute Carrier Family 7 Member 4

**FIGURE 8:**
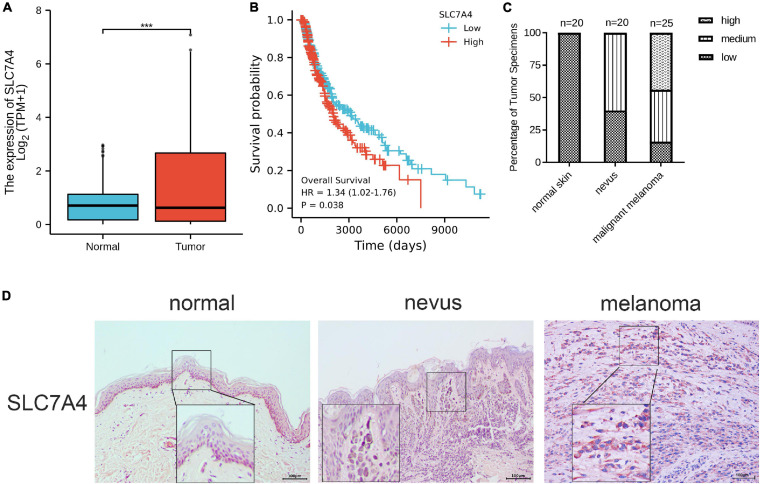
The expression signature, prognostic value, and preliminary validation of solute carrier family 7 member 4 (SLC7A4). **(A)** The expression of SLC7A4 was higher in SKCM than in normal skin tissues. **(B)** The overall survival analysis of SLC7A4 in SKCM. **(C,D)** IHC staining demonstrated that the expressions of SLC7A4 staining increased with the progression of the disease. Normal skin tissues (*n* = 20) showed the lowest expression, followed by benign nevi (*n* = 20), and malignant melanoma tissues (*n* = 25) showing the highest expression. IHC, immunohistochemistry. IHC stain, AEC, original magnification × 100, scale bar = 0.1 mm (inset, IHC stain, AEC, original magnification ×400).

The 90-gene classifier distribution in the three subtypes and the fold changes are plotted in the heat map shown in Figure 7A and Supplementary Table 5. Of these, SLC7A4 was not reported to be associated with melanoma among the 90 genes. Therefore, we performed an analysis and preliminary experimental validation of the role of SLC7A4 in SKCM. SLC7A4 was overexpressed in SKCM than in the adjacent normal skin tissues (Figure 8A). The OS was poorer in patients with high SLC7A4 expression than in those with low SLC7A4 expression (hazard ratio [HR] = 1.34 [1.02–1.76], *p* = 0.038, Figure 8B). SLC7A4 expression was determined in tissue samples using IHC staining. The results showed that melanoma tissues presented the highest expression of SLC7A4, followed by benign nevi, whereas normal skin tissues showed the lowest expression (Figures 8C,D). Generally, the intensity of SLC7A4 staining increased with the progression of melanoma. These results demonstrated that SLC7A4 plays a hub role in SKCM progression and, to some extent, proved the accuracy of this gene classifier.

## Discussion

Metabolic reprogramming is a key hallmark of cancer (Pavlova and Thompson, 2016). On the one hand, SKCM is characterized by its marked metabolic plasticity, and its development is associated with an important relationship between carcinogenesis and energy metabolism (Ruocco et al., 2019). On the other hand, metabolic reprogramming of melanoma alters the subset function of immune cells, enabling melanoma to evade the immune system. Thus, there is an urgent need to better understand the metabolic signatures of tumor heterogeneity in patients with melanoma to provide precise immunotherapy selection strategies and characterize the mechanisms of drug resistance. Herein, we performed a thorough classification of the metabolic profiles of SKCM specimens. Our findings revealed that SKCM could be categorized into three different metabolically relevant subtypes, and we verified the reproducibility of these subtypes in the GEO test set. Each subtype was characterized by different metabolic signatures, prognosis values, clinical parameters, tumor microenvironment features, immune cell infiltration, function and pathway enrichment, somatic copy number alterations, response to immunotherapy, and drug sensitivity. The C1 subtype displayed high metabolism levels, resembling the keratin subtype, with an advanced pathological stage and low immune infiltration. Furthermore, C1 had the worst prognosis and the shortest DFS among SKCM patients. In contrast, C2 was abundant in immune signatures with a relatively high expression of immune checkpoint genes and high immune and stromal scores, in line with the immune type and normal methylation type, demonstrating drug sensitivity to PD-1 inhibitors. This cluster was hardly engaged in metabolic signatures. The C3 subtype had the highest enrichment scores for carcinogenic pathways and the lowest enrichment scores for immune infiltration. It exhibited lower enrichment for metabolic signatures than C1 but greater enrichment than C2 for metabolic signatures. Overall, this research was undertaken to explore the metabolic landscape of SKCM and separately detect three clusters with different characteristics.

The results indicated that C1 had the most differential metabolic pathways, with 21 associated metabolic pathways all upregulated in this subtype. Therefore, we defined C1 as the metabolically active subtype. Several recent studies have focused on building a connection between endogenous tumor metabolism and immunotherapy. For example, recent studies have indicated that an increase in glycolytic metabolism in SKCM is linked to resistance to adoptive T-cell and immune checkpoint blockade therapies (Cascone et al., 2018). The hypermetabolic activation of C1 induces, on the one hand, nutrient depletion and hypoxia in the tumor microenvironment, thus, establishing metabolic competition between tumor cells and infiltrating immune cells. On the other hand, the active metabolism of SKCM in the tumor microenvironment leads to toxic concentrations of certain metabolites, including elevated concentrations of adenosine, kynurenine, ornithine, and reactive oxygen species, all of which markedly inhibit the anti-tumor immune response (Leone and Powell, 2020). The taxonomic classification model of SKCM reported in 2015 identified three transcriptomic clusters based on mRNA transcriptional gene function, namely, “immune,” “keratin,” and “MITF-low” (2015). Of the three subtypes, the keratin subtype had the poorest prognosis among patients with localized melanoma metastases and featured high expression of keratin-coding genes and metabolic genes in organ development, which was consistent with C1 having the poorest prognosis and characterized by enrichment gene signatures of keratin-related metabolic procedures. In addition, the clinical signature analysis showed that most patients in C1 were in the advanced pathological stage, followed by C3.

Several metabolic processes were upregulated in subclass C1, including amino acid metabolism, carbohydrate metabolism, and lipid metabolism. Enrichment results of metabolic signatures indicate the possibility that C1 could benefit from targeted metabolism therapy. Metabolic therapies targeting certain metabolic processes provide alternatives for chemoresistant patients. For instance, metformin can exert an anti-melanoma effect (Li et al., 2018) and promote combination treatment efficacy with anti-PD-1 and anti-cytotoxic T-lymphocyte-associated protein-4 (CTLA4) agents (Afzal et al., 2018; Cha et al., 2018). Identifying the potential beneficiaries of metabolic therapies has always proven to be challenging (Goodpaster and Sparks, 2017). Meanwhile, some key processes, such as glycolytic metabolism, hexosamine biosynthesis pathway, and glutathione metabolic pathways, were enriched in C3, suggesting that patients with this (HBP) are a shunt pathway of glycolysis and a key metabolic juncture in cancer cells. Recent studies have demonstrated that treatment with small molecule drugs that target HBP lead to increased infiltration of CD8 + T cells, sensitizing pancreatic tumors to anti-PD1 therapy and causing tumor regression and prolonged survival (Sharma et al., 2020). This implies that we may achieve precise targeting of C3 subclasses by targeting HBP in SKCM. This subcategory survey provides new clues to forecast potential candidates for metabolic therapies, which needs further verification in a large clinical cohort.

Tumor immune infiltration results showed that C2 had elevated levels of all immune cell lineages, indicating that it was in a highly immune-activating state. We believe that it contributes to the best prognosis of C2 among the three subtypes. C2 matches the immune subtype and is accompanied by the highest associated immune score. The immune subtype exhibited high expression of immune cell subsets (T cells, B cells, and NK cells), immune signaling molecules, and immune checkpoint-related genes, and patients with regional metastatic SKCM in the immune subtype had a better prognosis than the other two subtypes due to their active host immune response (2015). Furthermore, tumor microenvironment-related assessments revealed that C2 had higher immune and stromal scores. Research on SKCM classification based on TMB found that those with high immune scores had a favorable prognosis, and those with low immune scores had a poor prognosis (Hu et al., 2020), and these findings were consistent with the favorable prognosis of the immune-high C2 subtype in our study. Immune checkpoint blocking antibodies, such as pembrolizumab, nivolumab, and ipilimumab, which target PD-1 or CTLA-4, have considerably transformed the therapeutic landscape of SKCM in recent years (Schvartsman et al., 2019). C2 exhibited a higher expression of immune checkpoint genes, especially LAG3, CD274 (also known as PD-L1), PDCD1, CD247, CTLA4, PDCD1LG2, TNFRSF9, TNFRSF4, and TLR9, demonstrating that it shows promising sensitivity against anti-PD-1 therapy and other checkpoint inhibitors. The high expression enrichment score of PD-1 (CD274) may contribute to the susceptibility of C2 to anti-PD-1 therapy. This aligns with the results of the TIDE algorithm, in which C2 was sensitive to PD-1 immunotherapy. In contrast, immune checkpoint expression of fibrinogen-like protein 1 (FGL1) was highest in the C3 subtype, and FGL1 is the major LAG-3 functional ligand that acts independently of MHC-II (Wang et al., 2019). FGL1 represses antigen-specific T-cell activation and exerts a tumor immunosuppressive effect, and it is associated with poor prognosis and resistance to anti-PD-1 therapy.

The C3 subtype paralleled the MITF-low subtype of SKCM. The “MITF-low” cluster is marked with a low expression of genes associated with immune regulation (2015) and pigmentation markers, which is consistent with the low immune cell infiltration in C3. Meanwhile, studies have consistently reported that low MITF expression is an early resistance to multiple targeted drugs (Müller et al., 2014). Furthermore, the enrichment scores of C3 were significantly higher than those of C1 and C2 in most carcinogenic pathways, such as NRF2, PI3K, TGF-β, TP53, and Hippo, which accounts for the poor prognosis of patients in the C3 subgroup. In particular, activation of NRF2 inhibits the activity of the melanocyte lineage marker MITF and blunts the induction of innate immune responses in SKCM (Jessen et al., 2020). This is in line with the similarity of C3 with the MITF-low cluster. These data suggested that the C3 subclass was of high heterogeneity and might be refractory. Notably, previous studies demonstrated that the MITF-low subpopulation can be reversed by combining NK-κB inhibitors with SKCM resistance to BRAF inhibitors (Konieczkowski et al., 2014; Su et al., 2020), implying that the C3 subclass would best respond to the combination of NFK-κB inhibitors and BRAF inhibitors. C3 was also similar to the CpG island methylator phenotype (CIMP) (2015), one of the reported molecular subtypes of SKCM. Studies have shown that the CIMP pattern is implicated in the progression of the clinical stage of malignant melanoma (Tanemura et al., 2009). This is due to the fact that patients with SKCM of the C3 subtype are mostly at a higher pathological grade. Therefore, the poor prognosis in C3 may be attributed to the combined effects of low infiltration of immune cells, high enrichment of carcinogenesis pathways, low MITF expression, CIMP, and advanced pathological stage.

Skin cutaneous melanoma has an extremely high TMB due to ultraviolet (UV) mutagenesis (Lo et al., 2021), and these neoantigen burdens may alter T-cell responses in the tumor microenvironment (Hollern et al., 2019). Therefore, we examined whether copy number aberrations (deletions and amplifications), TMB, and neoantigens could be associated with the subtypes of SKCM. The results illustrated no statistically significant correlation between neoantigen burden and subtypes, with the TMB in C2 being the highest in the three types. Regarding copy number aberrations, C2 patients had fewer amplifications and deletions than C1 and C3. Our data revealed a high TMB in the C2 subclass, which is compatible with the result that the C2 subclass was sensitive to PD-1 treatment. Moreover, the number of somatic mutations was higher in C1 and C3 than in C2, which may lead to a poorer prognosis for C1 and C3. Notably, the high frequency of BRAF mutations in C2 and C3 implies that these two subtypes of patients may benefit from BRAF-targeted inhibitors (Menzer et al., 2019). In t-SNE analysis, C1 separated into two subpopulations, and some outliers of C1 were mixed with C3. We speculate that C1, which shows heterogeneity, is likely to be subdivided into two different subtypes, one of which is similar to C3 accompanied by similar stromal scores and mutation plots. In addition, C3 and C1 showed similar molecular patterns. Relatively higher metabolic signature level and worse prognosis, as well as lower abundance in immune signatures, distinguished C1 from C2. A more reasonable outcome will be attained if these C1 outliers are relabeled as C3.

Furthermore, we identified significant somatic mutation alteration sites in different subtypes according to GISTIC2.0. The number of loci and the degree of copy number variation of these genes were lower in C2 than in C1 and C3. In contrast, the number of amplification and deletion sites of the SKCM gene was higher in C3 than in C1 and C2. Furthermore, the magnitude of copy number variation showed the same trend. These results also showed a better prognosis of C2 than C1 and C3 and illustrated the association between the immune stromal infiltration of C1 and C3 and the occurrence of their tumor copy number variations. From the drug sensitivity analysis, we hypothesized that C2 may be more sensitive to chemotherapeutic agents than C1 and C3, suggesting that C2 may benefit from the combination of chemotherapy and immunotherapy. The GDSC drug sensitivity results indicated that two compounds, CGP-60474, a potent inhibitor of cyclin-dependent kinase (CDK), and bryostatin-1, a powerful protein kinase C (PKC) agonist, showed high drug sensitivity toward the three subtypes, representing promising chemotherapeutic drug candidates that target SKCM. The above discussion reveals that patients with the C2 subtype of SKCM may benefit from a combination of chemotherapy and immunotherapy. This study aimed to substantiate the need for personalized and precise treatment in clinical practice. The above discussion reveals that the three subtypes of SKCM may potentially benefit from the combination of chemotherapy and immunotherapy. This study aimed to provide supporting evidence for the availability of candidate target agents in clinical practice and achieve personalized precision therapy.

In the 90-gene classifier, many of the metabolic genes have been reported to play an important role in the inner metabolism and progression of SKCM, such as tyrosinase-related protein 1 (TYRP1) (Gilot et al., 2017) and OCA2 melanosomal transmembrane protein (OCN2) (Peterson et al., 2019), in particular, SLC7A4 of the solute carrier family of C1 to the transportation of cationic amino acids (Wolf et al., 2002). However, there have been no studies on SLC7A4 in SKCM. Therefore, we analyzed the expression signature of SLC7A4 and its prognostic value in TCGA-SKCM. We performed an IHC analysis of SLC7A4 from a clinical cohort to validate its role in SKCM. The results demonstrated that SLC7A4 was more highly expressed in melanoma tissues than in normal skin tissues, and the relatively higher expression of SLC7A4 presented a poor prognosis in SKCM patients, indicating that it could serve as a prognostic biomarker for SKCM. Moreover, the IHC results illustrated that the expression of SLC7A4 was proportional to the progression of melanoma. Therefore, SLC7A4 could be utilized as a therapeutic target for SKCM. These reports and experimental analyses reinforced the reliability of this gene classifier to a certain extent.

To the best of our knowledge, the present study is the first to generate a metabolic classification of SKCM that confirms the results of previous studies on SKCM subtypes while retaining their characteristics. Specifically, this classification matched the three subtypes from TCGA (keratin type, immune type, and MITF-low). C1 matched the keratin type and was highly metabolically active. C2 was the immune type, characterized by high immune infiltration, high expression of immune checkpoints, high immune and stromal scores, and favorable prognosis. We demonstrated for the first time that the C2 subtype corresponding to the immune type was prone to be a responder to PD-1 immunotherapy. C3 corresponded with MITF-low and was enriched in CpG island methylation. We also performed a preliminary experimental validation of the role of SLC7A4 in SKCM from the 90- gene classifier. Hence, the current study constitutes a novel demonstration of the presence of TCGA subtypes in TCGA-SKCM cohort and the GEO-SKCM cohort. Furthermore, this study not only validates the clinical significance of TCGA-SKCM classification but also reveals unexplored features, such as tumor microenvironment signatures and response to immunotherapy. This study provides new insights into the heterogeneity of SKCM in terms of the metabolic landscape by classifying SKCM into three clusters, active, intermediate, and depleted metabolic activity, which suggests possible therapeutic options for each subtype. However, we acknowledge several flaws in this study. First of all, more clinical data are needed to verify the reliability of this classification standard. Second, additional validation like cellular and molecular experiments of the classifier is required to prove the findings.

In conclusion, here, we proposed novel classifications of SKCM from a metabolic perspective with three subtypes, namely, metabolically active, intermediate, and depleted. C1 was closely associated with metabolic processes and had the worst prognosis, consistent with the keratin subtype. C2 exhibited higher immune infiltration, high immune and stromal scores, and sensitivity to PD-1 immune blockade agents, correlating with the immune type, and it had a favorable prognosis. C3 had an enriched carcinogenic pathway with a high degree of prognosis and relatively poor prognosis, and it was less metabolically active than C1 but more active than C2. Furthermore, the generated 90-gene classifier had a high predictive value for SKCM, and this classifier may help predict the prognosis of patients with SKCM more accurately and provide precise therapeutic approaches for these patients. Thus, our study further enhanced the recognition of the metabolic hallmarks of SKCM and contributed valuable new information regarding SKCM subtypes such that patients with this disease can receive personalized immunotherapy and more accurate prognosis prediction.

## Data Availability Statement

The datasets presented in this study can be found in online repositories. The names of the repository/repositories and accession number(s) can be found in the article/Supplementary Material.

## Ethics Statement

The studies involving human participants were reviewed and approved by the Ethics Committee of Foshan First People’s Hospital. The patients/participants provided their written informed consent to participate in this study.

## Author Contributions

RY and ZW conceived and designed the study. SZ, JL, ZW, RY, and YQ contributed to the data acquisition. JM, RG, and XP analyzed the data. ZW and SZ wrote the manuscript. All authors read and approved the final manuscript.

## Conflict of Interest

The authors declare that the research was conducted in the absence of any commercial or financial relationships that could be construed as a potential conflict of interest.

## Publisher’s Note

All claims expressed in this article are solely those of the authors and do not necessarily represent those of their affiliated organizations, or those of the publisher, the editors and the reviewers. Any product that may be evaluated in this article, or claim that may be made by its manufacturer, is not guaranteed or endorsed by the publisher.

## Footnotes

1
http://firebrowse.org/

2
http://software.broadinstitute.org/gsea/msigdb/index.jsp

3
https://cran.r-project.org/web/packages/ggplot2/index.html

4
http://kobas.cbi.pku.edu.cn

5
https://xenabrowser.net/datapages/

6
http://www.genepattern.org/

## Supplementary Material

Supplementary Figure 1 GO enrichment analysis of differential genes by Goplot package. GO enrichment of differential genes between C1 clusters and C2 and C3 clusters by the Goplot package, which is visualized as a taxonomic bubble plot **(A)** and a circle plot **(B)**. Venn diagram **(C)** of differential gene enrichment in “autophagy,” “protein binding,” “lysosome organization” three biological processes. Goplot package for GO enrichment of differential genes between C2 clusters and C1 and C3 clusters, and visualized as categorical bubble plots **(D)** and circle plots **(E)**. Venn diagram **(F)** of differential gene enrichment in the three biological processes “immune response,” “regulation of immune response,” “adaptive immune response.” GO enrichment of differential genes between C3 clusters and C1 and C2 clusters was carried out by the Goplot package and visualized in the form of classification bubble plots **(G)** and circle plots **(H)**. Venn diagram **(I)** of differential genes enriched in three biological processes: “protein binding,” “nucleotide-excision repair,” “DNA damage recognition,” and “poly(A) RNA binding.”

Supplementary Figure 2 KEGG pathway enrichment analysis was performed using KOBAS 3.0. The pathway figures depicted the common KEGG enrichment pathways for genes that are different between the three clustered subtypes and other subtypes. *p* < 0.05 pathways are considered significantly enriched. Apoptosis **(A)**, Cell cycle **(B)**, HIF-1 signaling pathway **(C)**, Human T-cell leukemia virus 1 **(D)**, Pathways in cancer infection **(E)**, PI3K-Akt signaling pathway **(F)**, red color stands for enriched genes with up-regulated expression.

Supplementary Figure 3 Mutation profiling of 90-gene classifier in the pan-cancer analysis and the TF-mRNA-miRNA network construction. **(A)** The gene mutation and gene copy number of 90-gene classifier in 32 TCGA pan-cancer databases were obtained from the cBioportal online database, and the analysis of the mutation was performed to illustrate the distribution proportion of each mutation type of this gene classifier in pan-cancer types, including gene mutation, fusion, amplification, deep deletion and multiple alterations in a bar chart. **(B)** Construction of potential transcription factor and miRNA (TF-mRNA-miRNA) interaction network for 90-gene classifier using Cytoscape software, with red representing transcription factors, blue representing mRNA, and green representing miRNA.

Supplementary Table 1 The information of clinical characteristics of TCGA-SKCM cohorts.

Supplementary Table 2 Independency test between meta cluster and other clinical information and previous classification in TCGA-SKCM cohorts.

Supplementary Table 3 Independency test between meta cluster and other clinical information in GEO-SKCM cohorts.

Supplementary Table 4 The ratio of driver-type oncogenic mutations based on TCGA-SKCM in each subtype.

Supplementary Table 5 The gene list of the 90 gene-classifier.

Supplementary Table 6 Analysis of GO enrichment of differential genes between subtypes C1 and C2 and C3 by Goplot package.

Supplementary Table 7 GO enrichment analysis of differential genes between subtypes C2 and subtypes C1 and C3 by Goplot package.

Supplementary Table 8 The GO enrichment analysis of differential genes between subtypes C3 and C1 and C2 by the Goplot package.

Supplementary Table 9 KEGG pathway enrichment analysis was performed using KOBAS 3.0 (*p* < 0.05), and the table showed the Top10 enriched KEGG pathways.

## Abbreviations

SKCM: skin cutaneous melanoma
ICI: immune checkpoint inhibitors
CTLA-4: cytotoxic T-lymphocyte-associated protein-4
GDSC: genomics of drug sensitivity in cancer
GEO: Gene Expression Omnibus
TCGA: The Cancer Genome Atlas
Tem: effective memory T cell
GSVA: gene set variation analysis
GSEA: gene set enrichment analysis
SLC7A4: solute carrier family 7 member 4
NMF: non-negative matrix factorization
CIMP: CpG island methylator phenotype
OS: overall survival.
