# Supplementary figures and images for: The Identification of the Metabolism Subtypes of Skin Cutaneous Melanoma Associated With the Tumor Microenvironment and the Immunotherapy
Source: Front Cell Dev Biol. 2021 Aug 12;9:707677. doi: 10.3389/fcell.2021.707677 (PMC8397464; doi:10.3389/fcell.2021.707677)

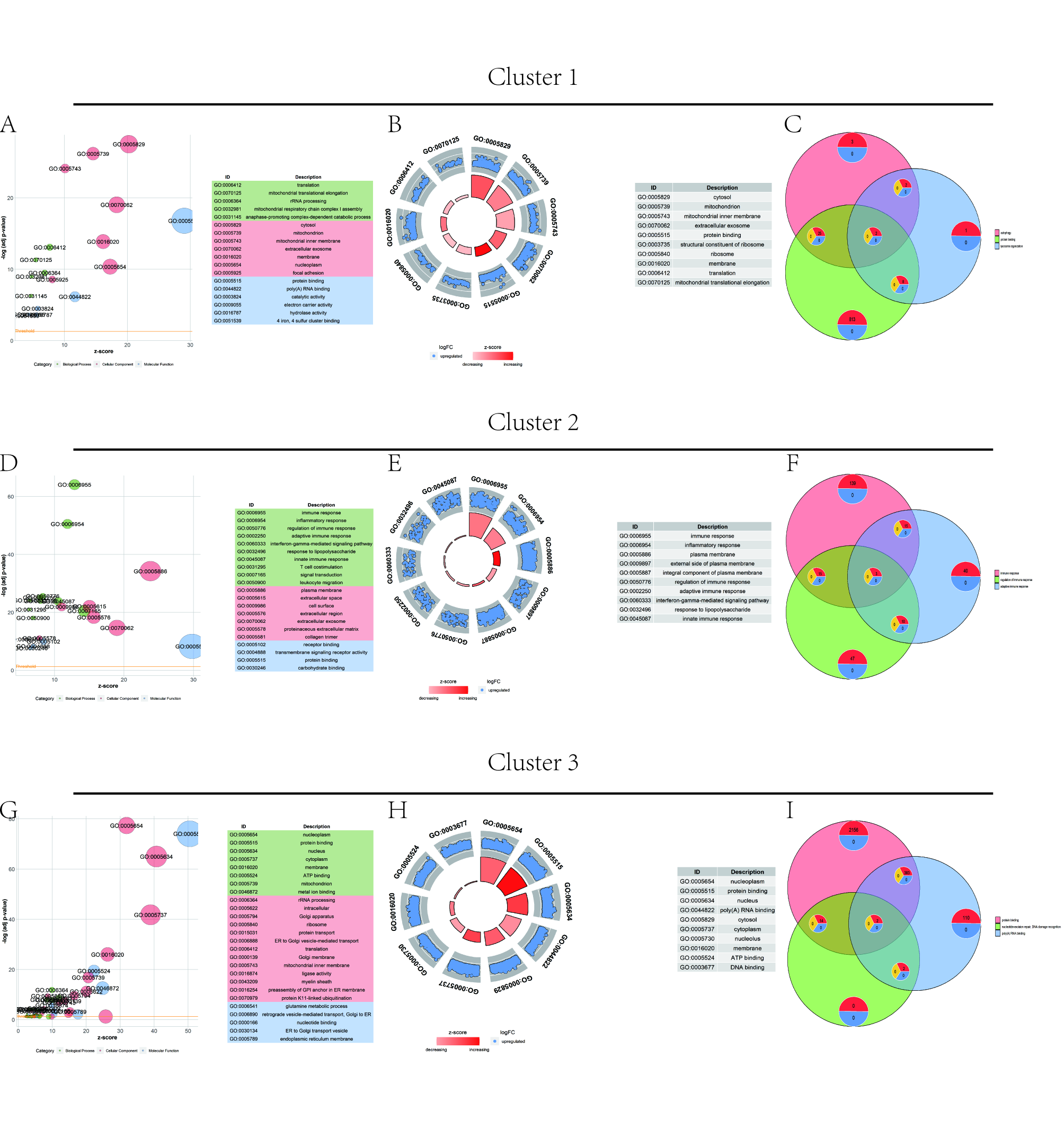

Supplement: Supplementary Figure 1 — GO enrichment analysis of differential genes by Goplot package. GO enrichment of differential genes between C1 clusters and C2 and C3 clusters by the Goplot package, which is visualized as a taxonomic bubble plot (A) and a circle plot (B). Venn diagram (C) of differential gene enrichment in “autophagy,” “protein binding,” “lysosome organization” three biological processes. Goplot package for GO enrichment of differential genes between C2 clusters and C1 and C3 clusters, and visualized as categorical bubble plots (D) and circle plots (E). Venn diagram (F) of differential gene enrichment in the three biological processes “immune response,” “regulation of immune response,” “adaptive immune response.” GO enrichment of differential genes between C3 clusters and C1 and C2 clusters was carried out by the Goplot package and visualized in the form of classification bubble plots (G) and circle plots (H). Venn diagram (I) of differential genes enriched in three biological processes: “protein binding,” “nucleotide-excision repair,” “DNA damage recognition,” and “poly(A) RNA binding.” [file Image_1.TIF]

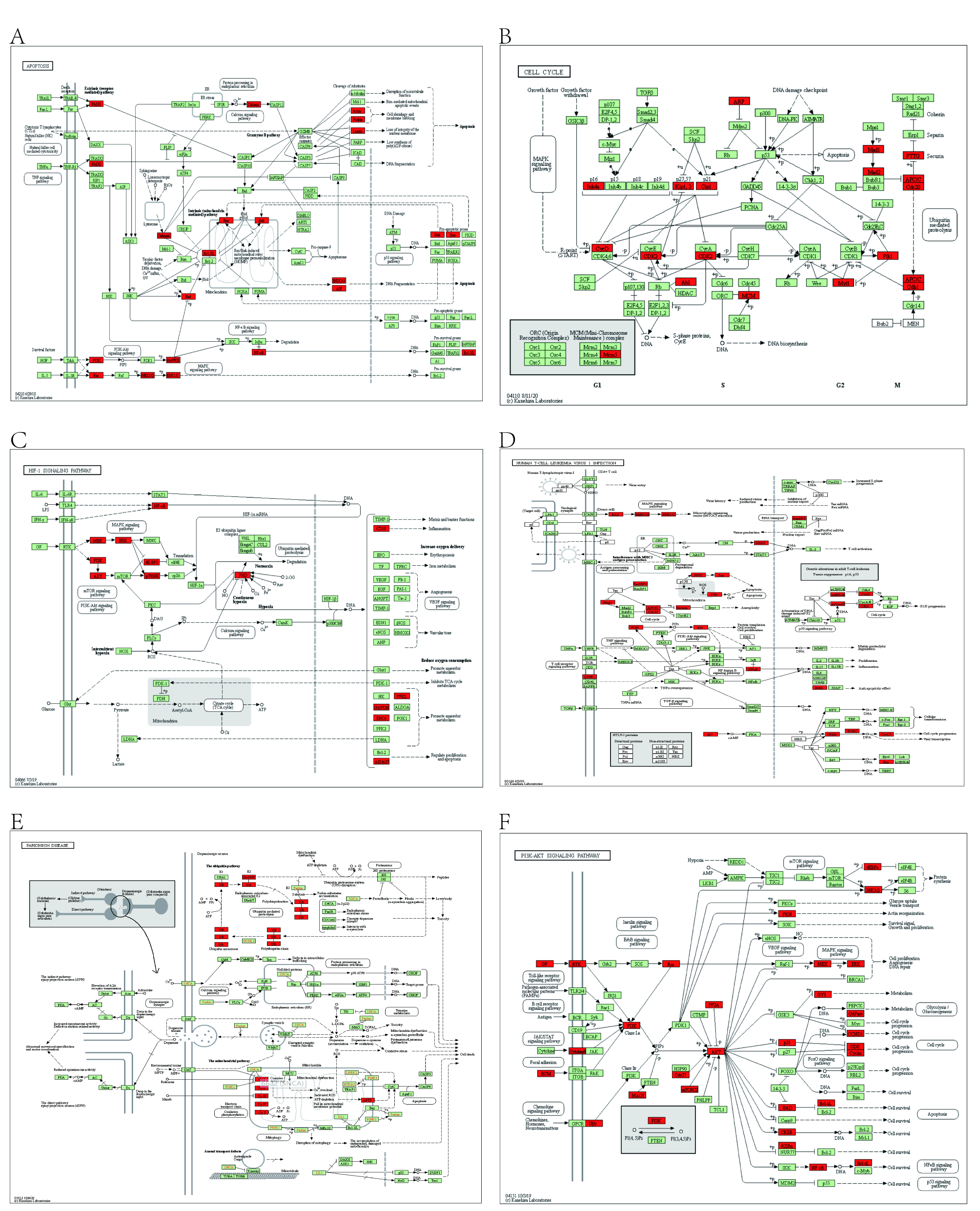

Supplement: Supplementary Figure 2 — KEGG pathway enrichment analysis was performed using KOBAS 3.0. The pathway figures depicted the common KEGG enrichment pathways for genes that are different between the three clustered subtypes and other subtypes. p < 0.05 pathways are considered significantly enriched. Apoptosis (A), Cell cycle (B), HIF-1 signaling pathway (C), Human T-cell leukemia virus 1 (D), Pathways in cancer infection (E), PI3K-Akt signaling pathway (F), red color stands for enriched genes with up-regulated expression. [file Image_2.TIF]

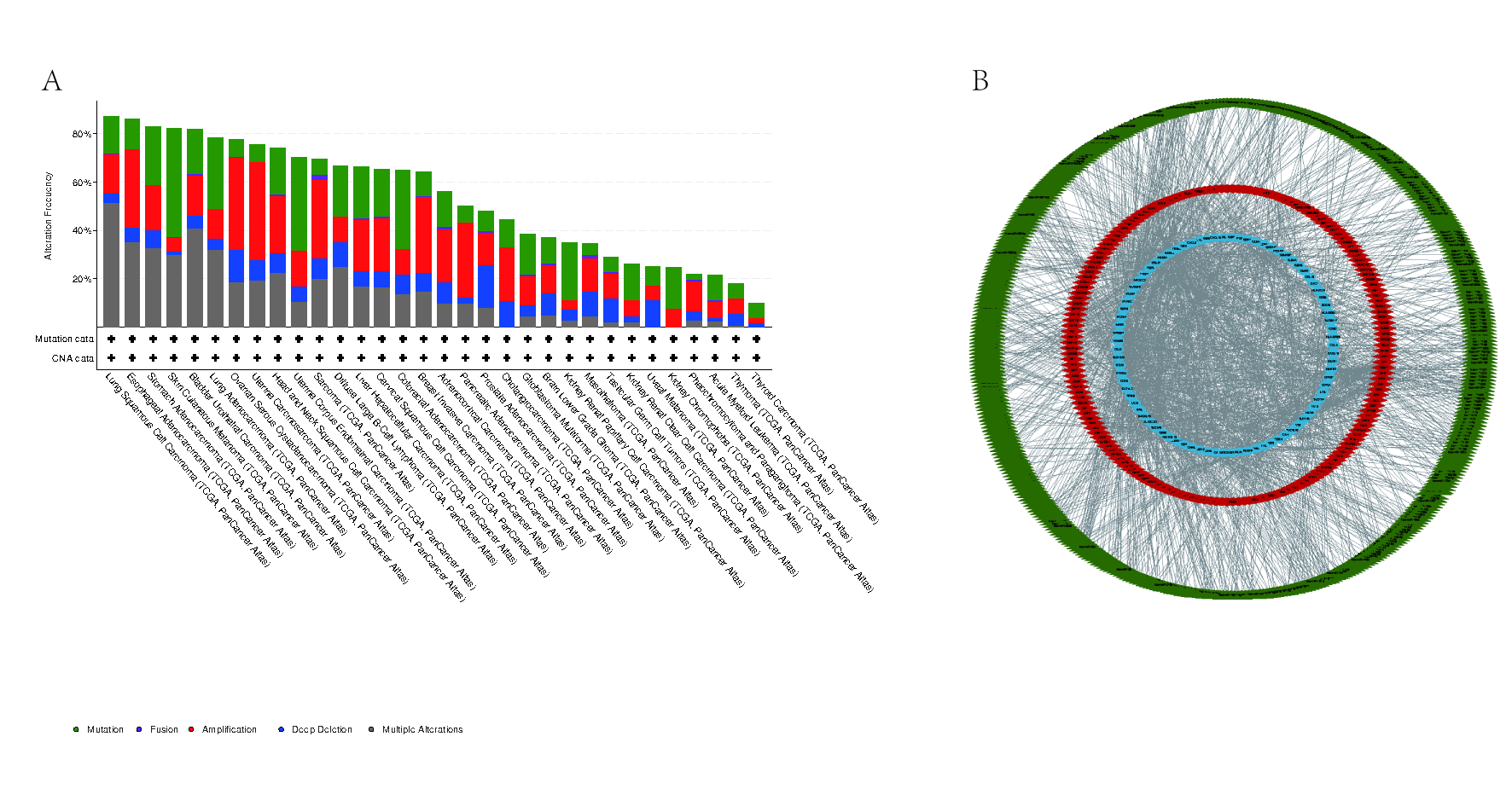

Supplement: Supplementary Figure 3 — Mutation profiling of 90-gene classifier in the pan-cancer analysis and the TF-mRNA-miRNA network construction. (A) The gene mutation and gene copy number of 90-gene classifier in 32 TCGA pan-cancer databases were obtained from the cBioportal online database, and the analysis of the mutation was performed to illustrate the distribution proportion of each mutation type of this gene classifier in pan-cancer types, including gene mutation, fusion, amplification, deep deletion and multiple alterations in a bar chart. (B) Construction of potential transcription factor and miRNA (TF-mRNA-miRNA) interaction network for 90-gene classifier using Cytoscape software, with red representing transcription factors, blue representing mRNA, and green representing miRNA. [file Image_3.TIF]
