# Supplementary material for: The Identification of the Metabolism Subtypes of Skin Cutaneous Melanoma Associated With the Tumor Microenvironment and the Immunotherapy
Source: Front Cell Dev Biol. 2021 Aug 12;9:707677. doi: 10.3389/fcell.2021.707677 (PMC8397464; doi:10.3389/fcell.2021.707677)
Supplement: Supplementary Table 1 — The information of clinical characteristics of TCGA-SKCM cohorts. [file Table_1.DOCX]

| Characteristic | levels | Overall |
| --- | --- | --- |
| n |  | 468 |
| T stage, n (%) | T1 | 41 (11.4%) |
|  | T2 | 78 (21.6%) |
|  | T3 | 90 (24.9%) |
|  | T4 | 152 (42.1%) |
| N stage, n (%) | N0 | 234 (56.9%) |
|  | N1 | 74 (18%) |
|  | N2 | 49 (11.9%) |
|  | N3 | 54 (13.1%) |
| M stage, n (%) | M0 | 416 (94.5%) |
|  | M1 | 24 (5.5%) |
| Pathologic stage, n (%) | Stage I | 76 (18.6%) |
|  | Stage II | 140 (34.2%) |
|  | Stage III | 170 (41.6%) |
|  | Stage IV | 23 (5.6%) |
| Gender, n (%) | Female | 179 (38.2%) |
|  | Male | 289 (61.8%) |
| Radiation therapy, n (%) | No | 381 (82.6%) |
|  | Yes | 80 (17.4%) |
| Race, n (%) | Asian | 12 (2.6%) |
|  | Black or African American | 1 (0.2%) |
|  | White | 445 (97.2%) |
| Age, n (%) | <=60 | 249 (54.1%) |
|  | >60 | 211 (45.9%) |
| Weight, n (%) | <=70 | 77 (30%) |
|  | >70 | 180 (70%) |
| Height, n (%) | < 170 | 117 (46.4%) |
|  | >=170 | 135 (53.6%) |
| BMI, n (%) | <=25 | 84 (33.7%) |
|  | >25 | 165 (66.3%) |
| Tumor tissue site, n (%) | Extremities | 195 (46.9%) |
|  | Trunk | 171 (41.1%) |
|  | Head and Neck | 37 (8.9%) |
|  | Other Specify | 13 (3.1%) |
| Melanoma ulceration, n (%) | No | 145 (46.6%) |
|  | Yes | 166 (53.4%) |
| Melanoma Clark level, n (%) | I | 6 (1.9%) |
|  | II | 18 (5.6%) |
|  | III | 76 (23.8%) |
|  | IV | 168 (52.7%) |
|  | V | 51 (16%) |
| Breslow depth, n (%) | <=3 | 184 (51.5%) |
|  | >3 | 173 (48.5%) |
| OS event, n (%) | Alive | 246 (53.4%) |
|  | Dead | 215 (46.6%) |
| DSS event, n (%) | Alive | 266 (58.5%) |
|  | Dead | 189 (41.5%) |
| PFI event, n (%) | Alive | 153 (33.2%) |
|  | Dead | 308 (66.8%) |
| Age, mean ± SD |  | 58.2 ± 15.75 |
