# Supplementary material for: The Identification of the Metabolism Subtypes of Skin Cutaneous Melanoma Associated With the Tumor Microenvironment and the Immunotherapy
Source: Front Cell Dev Biol. 2021 Aug 12;9:707677. doi: 10.3389/fcell.2021.707677 (PMC8397464; doi:10.3389/fcell.2021.707677)
Supplement: Supplementary Table 2 — Independency test between meta cluster and other clinical information and previous classification in TCGA-SKCM cohorts. [file Data_Sheet_1.PDF]

	Overall	C1	C2	C3	p	test
n	450	113	103	234		
ProteinCluster (%)					0.006	
PROT.ty	56 (29.6)	12 (32.4)	18 (32.7)	26 (26.8)		
PROT.ty	62 (32.8)	16 (43.2)	14 (25.5)	32 (33.0)		
PROT.ty	56 (29.6)	4 (10.8)	15 (27.3)	37 (38.1)		
PROT.ty	15 (7.9)	5 (13.5)	8 (14.5)	2 (2.1)		
MIRCluster (%)					<0.001	
MIR.type	75 (24.6)	20 (33.9)	26 (33.3)	29 (17.3)		
MIR.type	83 (27.2)	12 (20.3)	36 (46.2)	35 (20.8)		
MIR.type	78 (25.6)	18 (30.5)	8 (10.3)	52 (31.0)		
MIR.type	69 (22.6)	9 (15.3)	8 (10.3)	52 (31.0)		
MethTypes.201408 (%					<0.001	
CpG isla	79 (25.1)	15 (24.2)	7 (8.9)	57 (32.8)		
hyper-m	89 (28.3)	15 (24.2)	16 (20.3)	58 (33.3)		
hypo-m	81 (25.7)	19 (30.6)	16 (20.3)	46 (26.4)		
normal-l	66 (21.0)	13 (21.0)	40 (50.6)	13 (7.5)		
MUTATIONSUBTYPES					0.382	
BRAF_Hc	142 (47.3)	23 (39.0)	38 (48.7)	81 (49.7)		
NF1_Any	26 (8.7)	6 (10.2)	7 (9.0)	13 (8.0)		
RAS_Hot	87 (29.0)	18 (30.5)	18 (23.1)	51 (31.3)		
Triple_W	45 (15.0)	12 (20.3)	15 (19.2)	18 (11.0)		
TCGA_Subtype (%)					<0.001	
immune	161 (51.1)	27 (43.5)	67 (84.8)	67 (38.5)		
keratin	99 (31.4)	35 (56.5)	7 (8.9)	57 (32.8)		
MITF-lov	55 (17.5)	0 (0.0)	5 (6.3)	50 (28.7)		
OS = Deac	212 (47.1)	52 (46.0)	40 (38.8)	120 (51.3)	0.104	
pStage (%)					0.01	
Stage I	76 (18.8)	13 (12.4)	23 (26.4)	40 (18.9)		
Stage II	137 (33.9)	44 (41.9)	16 (18.4)	77 (36.3)		
Stage III	169 (41.8)	44 (41.9)	44 (50.6)	81 (38.2)		
Stage IV	22 (5.4)	4 (3.8)	4 (4.6)	14 (6.6)		
Gender =	279 (62.0)	71 (62.8)	56 (54.4)	152 (65.0)	0.178	
Age = >58	222 (49.3)	65 (57.5)	43 (41.7)	114 (48.7)	0.066	
	Overall	C1	C2	C3	p	test
n	289	93	98	98		
OS = Deac	150 (51.9)	54 (58.1)	38 (38.8)	58 (59.2)	0.006	
Gender =	174 (60.2)	58 (62.4)	57 (58.2)	59 (60.2)	0.839	
Age = >62	142 (49.3)	44 (47.3)	51 (52.6)	47 (48.0)	0.728	
