# Supplementary material for: The Identification of the Metabolism Subtypes of Skin Cutaneous Melanoma Associated With the Tumor Microenvironment and the Immunotherapy
Source: Front Cell Dev Biol. 2021 Aug 12;9:707677. doi: 10.3389/fcell.2021.707677 (PMC8397464; doi:10.3389/fcell.2021.707677)
Supplement: Supplementary Table 3 — Independency test between meta cluster and other clinical information in GEO-SKCM cohorts. [file Data_Sheet_2.PDF]

Independency test between metacluster and other clinical information in combined cohort

	Overall	C1	C2	C3	P test
n	289	93	98	98	
OS = Dead	150 (51.9)	54 (58.1)	38 (38.8)	58 (59.2)	0.006
Gender =	174 (60.2)	58 (62.4)	57 (58.2)	59 (60.2)	0.839
Age = >62	142 (49.3)	44 (47.3)	51 (52.6)	47 (48.0)	0.728
