# Supplementary material for: The Identification of the Metabolism Subtypes of Skin Cutaneous Melanoma Associated With the Tumor Microenvironment and the Immunotherapy
Source: Front Cell Dev Biol. 2021 Aug 12;9:707677. doi: 10.3389/fcell.2021.707677 (PMC8397464; doi:10.3389/fcell.2021.707677)
Supplement: Supplementary Table 4 — The ratio of driver-type oncogenic mutations based on TCGA-SKCM in each subtype. [file Data_Sheet_3.PDF]

	C1		C2		C3	
NRAS	27	0.28125	21	0.21	71	0.31982
BRAF	38	0.395833	63	0.63	121	0.545045
DDX3X	9	0.09375	9	0.09	21	0.094595
ARID2	16	0.166667	21	0.21	41	0.184685
MECOM	19	0.197917	30	0.3	62	0.279279
COL5A1	26	0.270833	33	0.33	69	0.310811
TP53	9	0.09375	21	0.21	42	0.189189
PTEN	6	0.0625	11	0.11	24	0.108108
KRAS	5	0.052083	2	0.02	5	0.022523
NF1	20	0.208333	22	0.22	43	0.193694
IDH1	8	0.083333	4	0.04	16	0.072072
PPP6C	4	0.041667	8	0.08	20	0.09009
HRAS	3	0.03125	3	0.03	7	0.031532
KIT	10	0.104167	5	0.05	22	0.099099
DACH1	9	0.09375	19	0.19	27	0.121622
RQCD1	2	0.020833	5	0.05	9	0.040541
RAC1	6	0.0625	9	0.09	16	0.072072
MAP2K1	5	0.052083	9	0.09	17	0.076577
BRD7	5	0.052083	5	0.05	8	0.036036
CDKN2A	13	0.135417	12	0.12	31	0.13964
CTNNB1	7	0.072917	6	0.06	16	0.072072
RB1	8	0.083333	5	0.05	12	0.054054
CDK4	4	0.041667	6	0.06	6	0.027027
GNA11	2	0.020833	7	0.07	11	0.04955
