# Supplementary material for: The Identification of the Metabolism Subtypes of Skin Cutaneous Melanoma Associated With the Tumor Microenvironment and the Immunotherapy
Source: Front Cell Dev Biol. 2021 Aug 12;9:707677. doi: 10.3389/fcell.2021.707677 (PMC8397464; doi:10.3389/fcell.2021.707677)
Supplement: Supplementary Table 5 — The gene list of the 90 gene-classifier. [file Data_Sheet_4.PDF]

ID	LogFC	Cluster
TYRP1	3.997493	Cluster 1
OCA2	3.6621024	Cluster 1
TSPAN10	2.5988354	Cluster 1
PAEP	2.4561358	Cluster 1
SLC7A4	2.1889245	Cluster 1
TRPM1	2.1089957	Cluster 1
GPR143	2.0101359	Cluster 1
SLC45A2	1.962466	Cluster 1
MLANA	1.8755778	Cluster 1
CA14	1.8717378	Cluster 1
GMPR	1.8583846	Cluster 1
TRIM63	1.8027831	Cluster 1
VGF	1.7550532	Cluster 1
DLL3	1.6868883	Cluster 1
SLC6A17	1.6498031	Cluster 1
MAL	1.6322201	Cluster 1
CDH3	1.6204952	Cluster 1
SLC16A6	1.5857662	Cluster 1
KRTAP19-1	1.579686	Cluster 1
TYR	1.5164881	Cluster 1
TFF3	1.4861308	Cluster 1
QPCT	1.4809012	Cluster 1
SLC7A5	1.4672749	Cluster 1
GSTP1	1.4513974	Cluster 1
LGI3	1.3870208	Cluster 1
FAM69B	1.3738372	Cluster 1
HES6	1.3689445	Cluster 1
BIRC7	1.348513	Cluster 1
LCE2A	1.3294721	Cluster 1
SCARB1	1.2710164	Cluster 1
CXCL9	3.0823183	Cluster 2
CXCL13	3.0400863	Cluster 2
CCL19	3.0284697	Cluster 2
PLA2G2D	3.0004169	Cluster 2
CCL21	2.866954	Cluster 2
GZMK	2.7805867	Cluster 2
CD3D	2.7363979	Cluster 2
LYZ	2.7155023	Cluster 2
CD79A	2.7056075	Cluster 2
CD2	2.6872386	Cluster 2
ADAMDEC1	2.6840883	Cluster 2
NKG7	2.6813074	Cluster 2
CD52	2.6669292	Cluster 2
GZMA	2.6630393	Cluster 2
LTB	2.6502096	Cluster 2
CXCL10	2.6098673	Cluster 2

CCL5	2.6097667	Cluster 2
CD8A	2.6072437	Cluster 2
CD48	2.5770746	Cluster 2
HLA-DRB1	2.5478981	Cluster 2
HLA-DQA1	2.5390348	Cluster 2
GBP5	2.5178216	Cluster 2
CD3E	2.5071422	Cluster 2
GZMB	2.4942055	Cluster 2
CD7	2.4461674	Cluster 2
HLA-DRB5	2.4456668	Cluster 2
UBD	2.4265082	Cluster 2
PTPRC	2.4114296	Cluster 2
CORO1A	2.3877734	Cluster 2
SLAMF6	2.3860278	Cluster 2
CDH19	1.6462524	Cluster 3
PTPRZ1	1.4342149	Cluster 3
B4GALT6	1.3385744	Cluster 3
MAGEC2	1.3347533	Cluster 3
PCDH7	1.3300782	Cluster 3
HMCN1	1.279808	Cluster 3
CDK6	1.2546537	Cluster 3
CHML	1.2358265	Cluster 3
GPR37	1.2172402	Cluster 3
SPRY2	1.1976607	Cluster 3
ELOVL2	1.1904934	Cluster 3
SEMA3C	1.1736203	Cluster 3
SLC5A3	1.1606346	Cluster 3
TRIM9	1.1562689	Cluster 3
TGDS	1.1478923	Cluster 3
WWTR1	1.1385907	Cluster 3
TRIM2	1.1307594	Cluster 3
CRISPLD1	1.1245452	Cluster 3
MBNL2	1.1214997	Cluster 3
NLGN1	1.1201925	Cluster 3
CDH2	1.1125485	Cluster 3
POSTN	1.110158	Cluster 3
MAP2	1.1055942	Cluster 3
SPRED1	1.1044373	Cluster 3
ITGAV	1.0844019	Cluster 3
PGAP1	1.0834454	Cluster 3
PEG10	1.0825193	Cluster 3
ITGB3	1.0821669	Cluster 3
SSFA2	1.0803436	Cluster 3
TNFRSF19	1.0800431	Cluster 3
