# Supplementary material for: The Identification of the Metabolism Subtypes of Skin Cutaneous Melanoma Associated With the Tumor Microenvironment and the Immunotherapy
Source: Front Cell Dev Biol. 2021 Aug 12;9:707677. doi: 10.3389/fcell.2021.707677 (PMC8397464; doi:10.3389/fcell.2021.707677)
Supplement: Supplementary Table 6 — Analysis of GO enrichment of differential genes between subtypes C1 and C2 and C3 by Goplot package. [file Table_6.docx]

**Table.S6 Analysis of GO enrichment of differential genes between subtypes C1 and C2 and C3 by Goplot package.**

| **Category** | **ID** | **Term** | **adj_pval** |
| --- | --- | --- | --- |
| BP | GO:0006412 | translation | 8.44E-14 |
| BP | GO:0070125 | mitochondrial translational elongation | 4.80E-12 |
| BP | GO:0070126 | mitochondrial translational termination | 4.01E-11 |
| BP | GO:0006364 | rRNA processing | 3.15E-10 |
| BP | GO:0032981 | mitochondrial respiratory chain complex I assembly | 1.08E-09 |
| BP | GO:0019083 | viral transcription | 1.72E-09 |
| BP | GO:0006120 | mitochondrial electron transport, NADH to ubiquinone | 1.93E-09 |
| BP | GO:0006614 | SRP-dependent cotranslational protein targeting to membrane | 9.96E-09 |
| BP | GO:0000184 | nuclear-transcribed mRNA catabolic process, nonsense-mediated decay | 4.56E-07 |
| BP | GO:0031145 | anaphase-promoting complex-dependent catabolic process | 5.36E-07 |
| CC | GO:0005829 | cytosol | 3.13E-28 |
| CC | GO:0005739 | mitochondrion | 6.74E-27 |
| CC | GO:0005743 | mitochondrial inner membrane | 7.94E-25 |
| CC | GO:0070062 | extracellular exosome | 9.45E-20 |
| CC | GO:0005840 | ribosome | 3.48E-15 |
| CC | GO:0016020 | membrane | 1.35E-14 |
| CC | GO:0005759 | mitochondrial matrix | 1.01E-11 |
| CC | GO:0005654 | nucleoplasm | 4.39E-11 |
| CC | GO:0005747 | mitochondrial respiratory chain complex I | 8.53E-10 |
| CC | GO:0005925 | focal adhesion | 2.72E-09 |
| MF | GO:0005515 | protein binding | 1.64E-17 |
| MF | GO:0003735 | structural constituent of ribosome | 8.44E-16 |
| MF | GO:0008137 | NADH dehydrogenase (ubiquinone) activity | 7.06E-08 |
| MF | GO:0044822 | poly(A) RNA binding | 6.98E-07 |
| MF | GO:0003954 | NADH dehydrogenase activity | 6.03E-06 |
| MF | GO:0003824 | catalytic activity | 3.12E-05 |
| MF | GO:0098641 | cadherin binding involved in cell-cell adhesion | 1.05E-04 |
| MF | GO:0009055 | electron carrier activity | 2.15E-04 |
| MF | GO:0016787 | hydrolase activity | 2.41E-04 |
| MF | GO:0051539 | 4 iron, 4 sulfur cluster binding | 3.25E-04 |
