# Supplementary material for: The Identification of the Metabolism Subtypes of Skin Cutaneous Melanoma Associated With the Tumor Microenvironment and the Immunotherapy
Source: Front Cell Dev Biol. 2021 Aug 12;9:707677. doi: 10.3389/fcell.2021.707677 (PMC8397464; doi:10.3389/fcell.2021.707677)
Supplement: Supplementary Table 7 — GO enrichment analysis of differential genes between subtypes C2 and subtypes C1 and C3 by Goplot package. [file Table_7.docx]

**Table.S7 GO enrichment analysis of differential genes between subtypes C2 and subtypes C1 and C3 by Goplot package.**

| **Category** | **ID** | **Term** | **adj_pval** |
| --- | --- | --- | --- |
| BP | GO:0006955 | immune response | 8.19E-65 |
| BP | GO:0006954 | inflammatory response | 3.10E-51 |
| BP | GO:0050776 | regulation of immune response | 2.43E-26 |
| BP | GO:0002250 | adaptive immune response | 7.53E-26 |
| BP | GO:0060333 | interferon-gamma-mediated signaling pathway | 6.96E-25 |
| BP | GO:0032496 | response to lipopolysaccharide | 1.18E-24 |
| BP | GO:0045087 | innate immune response | 1.57E-24 |
| BP | GO:0031295 | T cell costimulation | 1.03E-21 |
| BP | GO:0007165 | signal transduction | 3.01E-21 |
| BP | GO:0050900 | leukocyte migration | 8.34E-19 |
| CC | GO:0005886 | plasma membrane | 6.12E-35 |
| CC | GO:0009897 | external side of plasma membrane | 3.23E-31 |
| CC | GO:0005887 | integral component of plasma membrane | 2.08E-26 |
| CC | GO:0005615 | extracellular space | 8.17E-23 |
| CC | GO:0009986 | cell surface | 1.11E-22 |
| CC | GO:0005576 | extracellular region | 5.20E-19 |
| CC | GO:0070062 | extracellular exosome | 2.25E-15 |
| CC | GO:0045121 | membrane raft | 4.22E-13 |
| CC | GO:0005578 | proteinaceous extracellular matrix | 7.83E-12 |
| CC | GO:0005581 | collagen trimer | 1.86E-11 |
| MF | GO:0004872 | receptor activity | 3.46E-19 |
| MF | GO:0005102 | receptor binding | 1.14E-10 |
| MF | GO:0008009 | chemokine activity | 2.59E-10 |
| MF | GO:0004888 | transmembrane signaling receptor activity | 4.61E-09 |
| MF | GO:0005515 | protein binding | 5.41E-09 |
| MF | GO:0030246 | carbohydrate binding | 2.14E-08 |
| MF | GO:0042605 | peptide antigen binding | 2.25E-08 |
| MF | GO:0032395 | MHC class II receptor activity | 6.02E-08 |
| MF | GO:0005125 | cytokine activity | 1.75E-06 |
| MF | GO:0001618 | virus receptor activity | 1.82E-06 |
