# Supplementary material for: The Identification of the Metabolism Subtypes of Skin Cutaneous Melanoma Associated With the Tumor Microenvironment and the Immunotherapy
Source: Front Cell Dev Biol. 2021 Aug 12;9:707677. doi: 10.3389/fcell.2021.707677 (PMC8397464; doi:10.3389/fcell.2021.707677)
Supplement: Supplementary Table 8 — The GO enrichment analysis of differential genes between subtypes C3 and C1 and C2 by the Goplot package. [file Table_8.docx]

**Table.S8 The GO enrichment analysis of differential genes between subtypes C3 and C1 and C2 by the Goplot package.**

| **Category** | **ID** | **Term** | **adj_pval** |
| --- | --- | --- | --- |
| BP | GO:0006355 | regulation of transcription, DNA-templated | 5.14E-18 |
| BP | GO:0006351 | transcription, DNA-templated | 1.18E-17 |
| BP | GO:0006364 | rRNA processing | 1.41E-12 |
| BP | GO:0000398 | mRNA splicing, via spliceosome | 5.00E-11 |
| BP | GO:0016032 | viral process | 1.75E-10 |
| BP | GO:0000184 | nuclear-transcribed mRNA catabolic process, nonsense-mediated decay | 4.68E-10 |
| BP | GO:0016925 | protein sumoylation | 6.60E-10 |
| BP | GO:0019083 | viral transcription | 8.57E-10 |
| BP | GO:0006361 | transcription initiation from RNA polymerase I promoter | 2.95E-09 |
| BP | GO:0015031 | protein transport | 3.10E-09 |
| CC | GO:0005654 | nucleoplasm | 4.92E-78 |
| CC | GO:0005634 | nucleus | 1.05E-65 |
| CC | GO:0005829 | cytosol | 1.70E-51 |
| CC | GO:0005737 | cytoplasm | 1.70E-42 |
| CC | GO:0005730 | nucleolus | 1.09E-33 |
| CC | GO:0016020 | membrane | 1.04E-26 |
| CC | GO:0005739 | mitochondrion | 1.53E-17 |
| CC | GO:0005813 | centrosome | 1.68E-14 |
| CC | GO:0005622 | intracellular | 5.78E-12 |
| CC | GO:0005759 | mitochondrial matrix | 1.86E-10 |
| MF | GO:0005515 | protein binding | 9.14E-75 |
| MF | GO:0044822 | poly(A) RNA binding | 7.05E-55 |
| MF | GO:0005524 | ATP binding | 3.66E-20 |
| MF | GO:0003677 | DNA binding | 7.65E-19 |
| MF | GO:0003676 | nucleic acid binding | 2.39E-18 |
| MF | GO:0003723 | RNA binding | 2.23E-16 |
| MF | GO:0046872 | metal ion binding | 2.80E-13 |
| MF | GO:0004386 | helicase activity | 8.34E-10 |
| MF | GO:0004004 | ATP-dependent RNA helicase activity | 1.29E-09 |
| MF | GO:0003682 | chromatin binding | 7.20E-09 |
