# Supplementary material for: The Identification of the Metabolism Subtypes of Skin Cutaneous Melanoma Associated With the Tumor Microenvironment and the Immunotherapy
Source: Front Cell Dev Biol. 2021 Aug 12;9:707677. doi: 10.3389/fcell.2021.707677 (PMC8397464; doi:10.3389/fcell.2021.707677)
Supplement: Supplementary Table 9 — KEGG pathway enrichment analysis was performed using KOBAS 3.0 (p < 0.05), and the table showed the Top10 enriched KEGG pathways. [file Table_9.docx]

**Table.S9 KEGG pathway enrichment analysis was performed using KOBAS 3.0(p < 0.05), and the table showed the Top10 enriched KEGG pathways.**

| **Cluster** | **Term** | **ID** | **Input number** | **P-Value** |
| --- | --- | --- | --- | --- |
| C1 | Metabolic pathways | hsa01100 | 223 | 2.58E-69 |
| C1 | Oxidative phosphorylation | hsa00190 | 46 | 8.63E-27 |
| C1 | Huntington disease | hsa05016 | 50 | 3.06E-24 |
| C1 | Alzheimer disease | hsa05010 | 47 | 8.78E-24 |
| C1 | Ribosome | hsa03010 | 44 | 5.21E-23 |
| C1 | Parkinson disease | hsa05012 | 41 | 1.37E-21 |
| C1 | Non-alcoholic fatty liver disease (NAFLD) | hsa04932 | 39 | 2.39E-19 |
| C1 | Thermogenesis | hsa04714 | 47 | 3.61E-19 |
| C1 | Endocytosis | hsa04144 | 36 | 2.23E-11 |
| C1 | Retrograde endocannabinoid signaling | hsa04723 | 25 | 2.19E-09 |
| C2 | Cytokine-cytokine receptor interaction | hsa04060 | 92 | 1.11E-44 |
| C2 | Epstein-Barr virus infection | hsa05169 | 67 | 3.26E-34 |
| C2 | Hematopoietic cell lineage | hsa04640 | 51 | 8.17E-34 |
| C2 | Tuberculosis | hsa05152 | 61 | 1.06E-31 |
| C2 | Osteoclast differentiation | hsa04380 | 53 | 4.74E-31 |
| C2 | Pathways in cancer | hsa05200 | 96 | 6.88E-30 |
| C2 | Chemokine signaling pathway | hsa04062 | 60 | 1.01E-29 |
| C2 | Cell adhesion molecules (CAMs) | hsa04514 | 54 | 1.21E-29 |
| C2 | Viral protein interaction with cytokine and cytokine receptor | hsa04061 | 47 | 1.35E-29 |
| C2 | Natural killer cell mediated cytotoxicity | hsa04650 | 49 | 3.32E-27 |
| C3 | Herpes simplex virus 1 infection | hsa05168 | 135 | 8.71E-32 |
| C3 | Metabolic pathways | hsa01100 | 247 | 1.43E-28 |
| C3 | RNA transport | hsa03013 | 55 | 9.57E-17 |
| C3 | Protein processing in endoplasmic reticulum | hsa04141 | 54 | 3.45E-16 |
| C3 | Ubiquitin mediated proteolysis | hsa04120 | 48 | 1.76E-15 |
| C3 | Pathways in cancer | hsa05200 | 102 | 4.70E-15 |
| C3 | Cell cycle | hsa04110 | 44 | 1.79E-14 |
| C3 | Proteoglycans in cancer | hsa05205 | 52 | 3.77E-12 |
| C3 | mRNA surveillance pathway | hsa03015 | 34 | 5.10E-12 |
| C3 | PI3K-Akt signaling pathway | hsa04151 | 71 | 1.28E-11 |
